# Bridging of *Neisseria gonorrhoeae* lineages across sexual networks in the HIV pre-exposure prophylaxis era

**DOI:** 10.1038/s41467-019-12053-4

**Published:** 2019-09-05

**Authors:** Deborah A. Williamson, Eric P. F. Chow, Claire L. Gorrie, Torsten Seemann, Danielle J. Ingle, Nasra Higgins, Marion Easton, George Taiaroa, Yonatan H. Grad, Jason C. Kwong, Christopher K. Fairley, Marcus Y. Chen, Benjamin P. Howden

**Affiliations:** 1 Microbiological Diagnostic Unit Public Health Laboratory, Department of Microbiology and Immunology, The University of Melbourne at The Peter Doherty Institute for Infection and Immunity, Melbourne, Australia; 2 Melbourne Sexual Health Centre, Alfred Health, Carlton, VIC Australia; 3 Central Clinical School, Monash University, Melbourne, Australia; 4 Melbourne Bioinformatics Group, Melbourne, VIC Australia; 5 National Centre for Epidemiology and Population Health, The Australian National University, Canberra, Australia; 6 Victorian Department of Health and Human Services, Melbourne, Australia; 7 Department of Immunology and Infectious Diseases, Harvard T. H. Chan School of Public Health, Boston, MA USA

**Keywords:** Antimicrobial resistance, Infectious-disease epidemiology, Policy and public health in microbiology, Risk factors

## Abstract

Whole genome sequencing (WGS) has been used to investigate transmission of *Neisseria gonorrhoeae*, but to date, most studies have not combined genomic data with detailed information on sexual behaviour to define the extent of transmission across population risk groups (bridging). Here, through combined epidemiological and genomic analysis of 2,186*N. gonorrhoeae* isolates from Australia, we show widespread transmission of *N. gonorrhoeae* within and between population groups. We describe distinct transmission clusters associated with men who have sex with men (MSM) and heterosexuals, and men who have sex with men and women (MSMW) are identified as a possible bridging population between these groups. Further, the study identifies transmission of *N. gonorrhoeae* between HIV-positive and HIV-negative individuals receiving pre-exposure prophylaxis (PrEP). Our data highlight several groups that can be targeted for interventions aimed at improving gonorrhoea control, including returning travellers, sex workers, and PrEP users.

## Abstract (web summary)

Here, Williamson *et al*. combine epidemiological and genomic analysis of 2,186 *Neisseria gonorrhoeae* isolates from Australia and show that men who have sex with men and women are a possible ‘bridging’ population between men who have sex with men and heterosexuals.

## Introduction

Gonorrhoea is a common sexually transmitted infection (STI) caused by *Neisseria gonorrhoeae*, and represents a major health problem globally. The emergence and spread of antimicrobial-resistant (AMR) *N. gonorrhoeae* has been deemed an urgent threat to public health^1^. Untreated, gonorrhoea can lead to severe sequelae, notably pelvic inflammatory disease, ectopic pregnancy and infertility in women, and can promote the transmission of HIV^2^.

Similar to the United States (US)^3^ and United Kingdom (UK)^4^, the incidence of gonorrhoea in Australia has increased markedly over the past 5 years^3–6^. Between 2012 and 2017, the annual incidence of gonorrhoea notifications in Australia rose from 61.1 per 100,000 population to 115.4 per 100,000 population, an alarming increase of 89%^7^. Although the increase in incidence may partly be due to increased diagnostic sensitivity of nucleic acid amplification tests (NAATs), a true increase in disease is supported by concurrent increases in other bacterial STIs, such as chlamydia and syphilis^5,8^.

For decades, prevention of gonorrhoea has relied on public health measures, such as condom use, educational messaging, and screening to detect asymptomatic carriage of *N. gonorrhoeae*. Despite these measures, the incidence of gonorrhoea is increasing, predominantly in men who have sex with men (MSM), but also more recently in heterosexual populations^4,8^. The reasons for this are not clear, although a number of factors may contribute. For example, the use of online dating applications has been associated with outbreaks of bacterial STIs among MSM^9,10^. Further, the introduction of pre-exposure prophylaxis (PrEP) for HIV has been associated with a decrease in condom use amongst MSM, and in some settings, an increase in STIs^11,12^. Finally, increasing travel may lead to increased importation of *N. gonorrhoeae* (particularly AMR strains) from areas with a high prevalence of STIs, with subsequent endemic local transmission^13,14^. Improved knowledge of factors that promote transmission could enable public health interventions to be directed towards those at highest risk of disease.

Recently, whole genome sequencing (WGS) has been used to provide insights into the local and global emergence and transmission of gonorrhoea, including AMR lineages^15–19^. To date, however, these studies have not focused on assessing transmission across population risk groups (bridging), utilising individual-level sexual behavioural data. Here, we hypothesise that combining individual-level data with genomic analyses will uncover widespread transmission of *N. gonorrhoeae* within and between distinct sexual risk networks, and that this could identify priority areas for public health interventions. Through detailed genomic analysis of all *N. gonorrhoeae* isolates detected in the state of Victoria, Australia in 2017, we reveal extensive bridging of lineages across groups with differing epidemiological risk factors, including sexual behaviour, overseas travel, HIV status and sex work. We also demonstrate circulation of an emerging azithromycin-resistant clone, associated with a mosaic *mtr* locus, representing a largely under-recognised mechanism of azithromycin resistance. Our findings represent the most comprehensive genomic analysis of *N. gonorrhoeae* from the Southern Hemisphere to date and highlight the need for new and enhanced public health interventions to control this 21st century epidemic of gonorrhoea.

## Results

### Epidemiological characteristics of patients

Between January 1, 2017 and December 31, 2017, there were 7309 gonorrhoea notifications in Victoria (Supplementary Fig. 1); of these notifications 5941 (81.3%) were from males and 1368 (18.7%) were from females. From these 7309 notifications, 2055 patients (28.1%) had associated *N. gonorrhoeae* isolates, and, including multiple isolates from the same patients, a total of 2186 isolates underwent WGS (Table 1). Of the sequenced isolates, 882 were from the urethra, 632 from the rectum, 386 from the pharynx, and 227 from the cervix (Table 1). The majority of individuals with *N. gonorrhoeae* isolates were males (1777/2059; 86.3%), and of those males with reported sexual risk factor (1571/1777; 88.4%), most identified as MSM (1329/1571; 84.6%).

**Table 1:** Characteristics of *Neisseria gonorrhoeae* patients and isolates included in this study

Characteristic	Number (% of total)
*Sex*	
Male	1777 (86.3)
Female	270 (13.1)
Other	8 (0.4)
*Median age (years) at diagnosis (IQR)*	
All	29 (25–37)
Males	30 (25–37)
Females	27 (23–37)
Other	30 (27–34)
*Referring clinic*	
Sexual health clinic	1315 (64.0)
Males	1242
Primary care	646 (31.4)
Males	471
Hospital/outpatient	94 (4.6)
Males	64
*Sexual exposure (self-reported)*	
MSM	1329 (64.7)
MSMO	1282 (62.4)
MSMW	47 (2.3)
Heterosexual males	242 (11.8)
Heterosexual females	270 (13.1)
Not reported/unknown	214 (10.4)^a^
*HIV status*	
HIV-positive	221 (10.8)
MSM	214
Heterosexual males	6
Females	1
HIV-negative	1520 (74.0)
MSM	1093
PrEP	315 (28.8)^b^
Heterosexual males	205
Females	191
Unknown	314
*Isolate site*	
Urethra	882 (40.3)
Rectum	632 (28.9)
Pharynx	386 (17.7)
Cervix	227 (10.4)
Urine	32 (1.5)
Joint fluid	6 (0.3)
Eye	7 (0.3)
Unknown	14 (0.6)

*MSM* men who have sex with men, *MSMO* men who have sex with men only, *MSMW* men who have sex with men and women, *PrEP* pre-exposure prophylaxis

^a^All male individuals

^b^% of all MSM included in this study

The median age of patients was 29 years (interquartile range 25–37 years); most (953/2055; 46.4%) were in the 20–29-year age group (Supplementary Fig. 2). Approximately two-thirds of patients were from sexual health clinics (1315/2055; 64.0%), and approximately 11% of patients were HIV-positive (221/2055; 10.8%). Of the 1093 HIV-negative MSM, 315 (28.8%) reported PrEP use (Table 1).

One hundred and four patients had two or more isolates cultured ≥14 days apart over the study period, with a median of 122 days between first and last isolates (range 18–351 days). In 88 of these patients (88%), the isolates associated with these infections differed by more than 100 SNPs, consistent with new infections (Supplementary Fig. 3A). Compared to the 1951 patients with only one infection, these 88 individuals with two or more infections were significantly more likely to be MSM (*P* < 0.01, chi-squared test), HIV-positive (*P* < 0.01, chi-squared test), or PrEP users (*P* < 0.01, chi-squared test) (Supplementary Table 1). Of the 16 patients whose isolates were cultured ≥14 days apart, but differed by <100 SNPs, nine patients had repeat isolates that belonged to the same transmission cluster as their initial isolate (median duration between isolates 66 days, range 18–201 days), potentially indicating reinfection from the same sexual network (Supplementary Fig. 3B). Overall, there were 13 patient pairs (11 MSM and 2 heterosexual) that identified each other as sexual partners, with a median of three SNPs (range 1–10 SNPs) between isolates from each pair, similar to a previous study by Kwong et al.^20^, that identified a median pairwise SNP distance of 5 (IQR 4–9) between *N. gonorrhoeae* isolates from 34 male couples in a known sexual partnership.

Amongst the 2186 isolates, 162 known *N. gonorrhoeae* multi-antigen sequence types (NG-MAST types) were identified (Supplementary Data 1). A total of 609 isolates (27.9% of the dataset) did not have a known NG-MAST type. The six most common STs were 5441 (11.2%); 5 (8.1%); 5802 (7.6%); 4186 (6.6%), 5624 (5.6%), and 2992 (5.3%), and the median pairwise SNP distance between isolates within these STs ranged from 4 to 1130 SNPs (Supplementary Fig. 4). NG-MAST types were distributed across the phylogeny and did not necessarily correlate with degree of relatedness as identified by WGS (Supplementary Fig. 5).

### Transmission analysis and association with risk groups

**Fig. 1:**
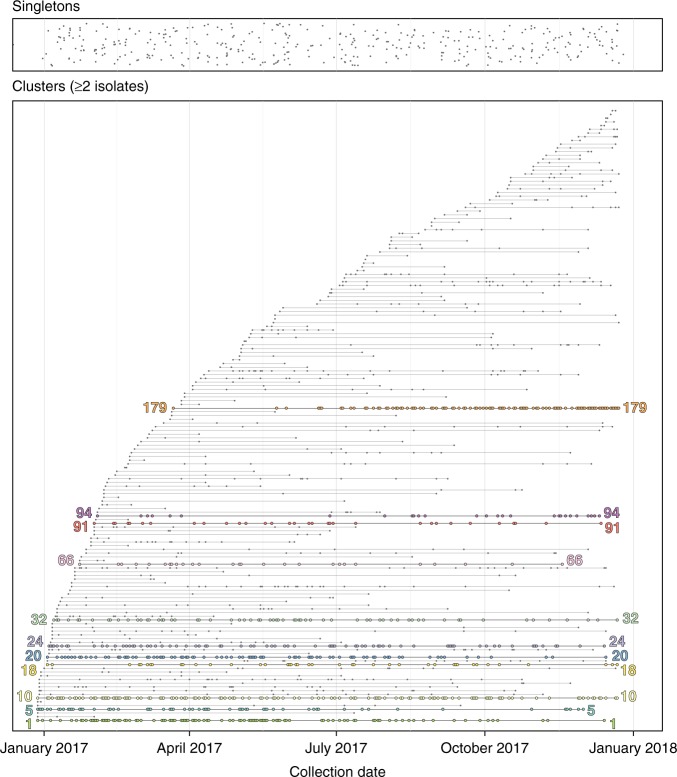
Clusters of *Neisseria gonorrhoeae* isolates in Victoria, Australia, 2017. Isolates are classified according to whether they are single isolates (top box) or in a cluster of ≥2 isolates (bottom box). Each cluster contains all individuals with isolates related by ≤10 single nucleotide polymorphisms using hierarchical single-linkage clustering. Major clusters (comprising >30 individuals) are highlighted on the timeline in the bottom box. Each dot represents an isolate from an individual, and each cluster is plotted along a horizontal line, representing date of sample collection

Hierarchical single-linkage clustering was performed on the pairwise SNP differences between isolates. To define a putative transmission event, we chose the maximum pairwise SNP threshold (10 SNPs, described above) between isolates from epidemiologically linked pairs (i.e. individuals who identified each other as sexual contacts and attended the clinic on the same day for testing). We deliberately elected not to remove recombination from the dataset as our primary aim was not to assess the emergence and evolution of specific lineages, but rather to identify potential transmission events within a geographically and temporally restricted dataset. For inclusion in a cluster, isolates from individuals had to be related to one or more other isolates in the cluster. Excluding intra-individual isolates and using our epidemiologically corroborated single-linkage 10 SNP threshold to define a possible transmission event between individuals, we identified 161 clusters of two or more related isolates, representing 83.2% (1819/2186 isolates) of the dataset (Fig. 1). The median size of each cluster was three patients (range 2–181 patients), and the median time from first to last patient in each cluster was 102 days (range 0–362 days). In general, larger clusters tended to be of longer duration (Supplementary Fig. 6).

**Fig. 2:**
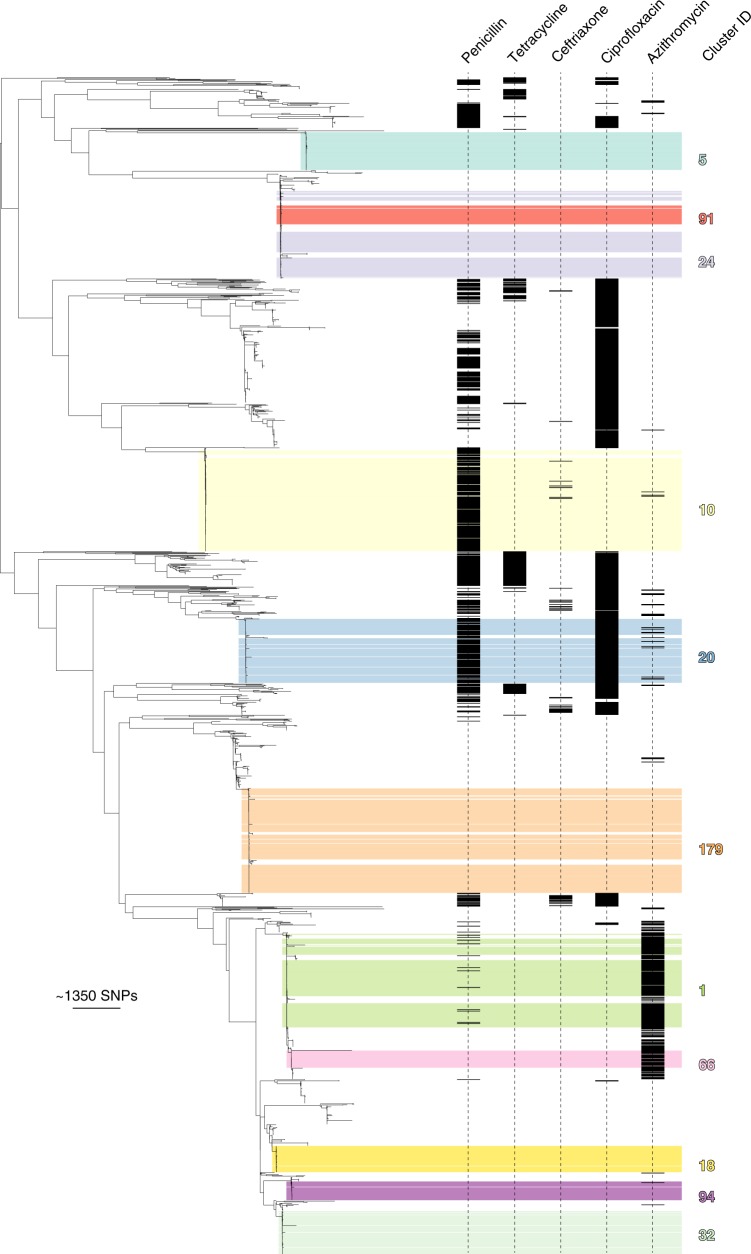
Population structure of 2186 *N. gonorrhoeae* isolates included in this study. The mid-point rooted maximum-likelihood tree (derived from 45,145 core SNPs) is plotted on the left. Phenotypic resistance (or decreased susceptibility for ceftriaxone) profiles are shown on the right, and major clusters are highlighted in colour. The scale bar represents the single nucleotide polymorphism (SNP) distance

**Fig. 3:**
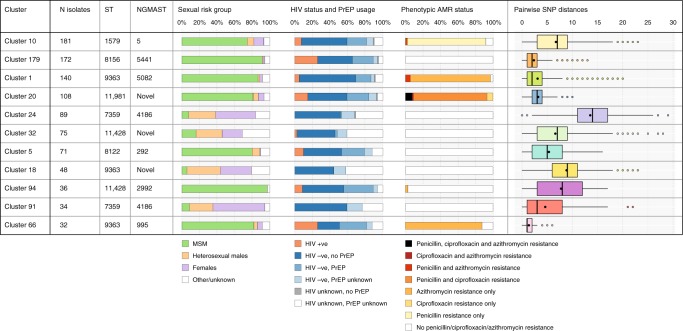
Associations of major *Neisseria gonorrhoeae* clusters with epidemiological risk factors and antimicrobial susceptibility profiles. Major clusters are defined as those containing 30 or more cases. The median pairwise single nucleotide polymorphism (SNP) distance represents the overall diversity within each cluster, with each cluster containing isolates that are related by ≤10 SNPs using a hierarchical single-linkage clustering approach. Box plots indicate median and interquartile range (IQR), with the whiskers representing the highest and lowest values within 1.5 × IQR of the upper and lower quartiles, and the dots representing outlier values. *Abbreviations*: MLST multilocus sequence type, NG-MAST *Neisseria gonorrhoeae* multi-antigen sequence types, *MSM* men who have sex with men, *PrEP* pre-exposure prophylaxis, *AMR* antimicrobial resistance, *SNP* single nucleotide polymorphism

**Fig. 4:**
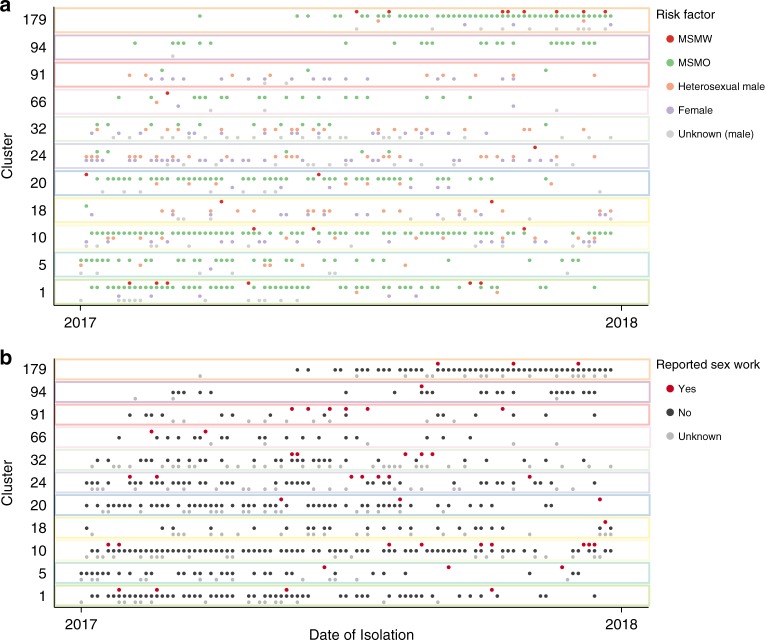
Timeline of *Neisseria gonorrhoeae* isolates from the 11 major clusters. The figure is stratified by **a** reported sexual behaviour and **b** reported sex work. The *x*-axis represents time, and each dot within the plots represents a single isolate. Data points are coloured by risk factor. *Abbreviations*: MSMO men who have sex with men only, *MSMW* men who have sex with men and women

To identify demographic associations with dominant clusters of *N. gonorrhoeae* in our study, we linked epidemiological characteristics with clusters that contained 30 or more individuals (a total of 11 clusters) (Figs. 2 and 3). Clusters were defined as predominantly MSM-related or heterosexual-related depending on whether MSM or heterosexuals were associated with 70% or more of isolates in that cluster (Supplementary Table 2). There were clear differences in sexual risk factors reported by individuals in different clusters. For example, the proportion of men who had sex with men only (MSMO) in each cluster varied from 2.6% (cluster 18) to 100% (cluster 94) (Supplementary Table 2). In general, clusters that were heterosexual-related had a lower median age than MSM-related clusters (Supplementary Table 2). Apart from cluster 94, which was exclusively associated with MSMO, all major clusters contained at least one female patient and in cluster 10 (the largest MSM-related cluster), there were 21 females, highlighting bridging between MSM and heterosexuals (Supplementary Table 2). The potential for bridging was further supported by the presence of men who have sex with men and women (MSMW) in seven of the major clusters (Fig. 4a).

All major clusters had at least one case who identified as a sex worker—this was more common in heterosexual-related clusters (Supplementary Table 2 and Fig. 4b). Female sex workers were more common in heterosexual-related clusters, whereas male sex workers were more common in MSM-related clusters (Supplementary Table 2). Compared to non-sex-workers, sex workers were more likely to have oropharyngeal infections (313/1685; 18.6% vs. 39/86; 45.3%, respectively; *P* < 0.01, chi-squared test).

Importantly, we also observed eight dominant clusters that contained both HIV-positive and HIV-negative MSM indicating disassortative sexual mixing and gonorrhoea transmission between these groups (Fig. 3). Clusters 179 and 66 were particularly associated with HIV-positivity and PrEP use amongst MSM, and these were also the clusters with the highest median age (Supplementary Table 2). PrEP use was reported by more than 20% of HIV-negative individuals in major MSM-related clusters (Supplementary Table 2 and Fig. 3). In addition, the median cluster size amongst PrEP users was significantly higher than that amongst HIV-negative non-PrEP users (Supplementary Fig. 7A). Further, when clusters were assessed over time, there was evidence of sustained transmission of some clusters between HIV-positive and HIV-negative individuals, including those individuals not taking PrEP (Supplementary Fig. 7B).

Data on recent sexual contact overseas were available from 1712 patients. Patients that reported recent sex overseas were significantly more likely to have non-cluster-associated infections than patients who had acquired their infections locally (52/281; 18.5% non-cluster-associated infection vs. 44/1431; 3.1% in a cluster; *P* < 0.001, chi-squared test) (Supplementary Table 3). Of the 31 clusters containing patients who reported recent sex overseas, the first isolate in 13 (42%) of these clusters was from a travel-associated case (Supplementary Fig. 8). In addition, there was a significantly higher proportion of heterosexual males amongst patients with overseas-associated infections compared to patients with locally acquired infections (31/96; 32.3% overseas-associated vs. 192/1616; 11.9% locally acquired; *P* < 0.01, chi-squared test). Further, isolates from patients acquiring their infections overseas were more likely to be resistant to penicillin, tetracycline and ciprofloxacin, and less resistant to azithromycin compared to patients with locally acquired infections (*P* < 0.01, chi-squared tests) (Supplementary Table 3).

### Co-circulation of AMR *N. gonorrhoeae* lineages

All 2186 isolates underwent susceptibility testing. Low-level azithromycin resistance (≥1 mg/L) was identified in 13.7% of isolates, resistance to penicillin and ciprofloxacin was identified in 29.5% and 30.8% of isolates, respectively, and only 2.1% of isolates displayed decreased susceptibility to ceftriaxone (Supplementary Table 4). Phenotypic and genotypic resistance profiles are detailed in Supplementary Data 1, and are shown relative to the phylogeny in Fig. 2.

Low-level azithromycin resistance was significantly higher in isolates from MSM than isolates from females or heterosexual males (17.0% vs. 5.4% vs. 3.6%, respectively; *P* < 0.01, chi-squared test) (Supplementary Table 4), and was not present in any clusters that contained exclusively heterosexual cases. Of note, 254/297 (85.5%) of low-level azithromycin isolates belonged to MLST 9363; within ST9363, there were 13 clusters, the largest of which were cluster 1 and cluster 66 (Supplementary Data 1 and Fig. 3). All isolates in clusters 1 and 66 harboured a mosaic *mtrD* allele, identical to the *Neisseria meningitidis*-like mosaic *mtrD* region recently described by Wadsworth et al. in the United States^21^, and to isolates previously identified in the United Kingdom (Supplementary Fig. 9)^18^, and more recently in the United States^22^. To further contextualise our data, we undertook combined analysis of the 388 MLST 9363 isolates in our study, and publicly available MLST 9363 isolates (151 isolates) from a recent US Gonococcal Surveillance Isolate Project (GISP) study conducted between 2014 and 2016^22^. 381 isolates (292 from Australia and 89 from the United States) contained an identical mosaic *mtrD*, and an identical mosaic *mtrR* gene (Supplementary Data 2 and Supplementary Fig. 10).

## Discussion

To our knowledge this is the first study to combine WGS of *N. gonorrhoeae* with comprehensive individual-level sexual behavioural data, providing a detailed picture of the transmission of distinct gonococcal strains between MSM, heterosexual males and females, HIV-positive MSM, HIV-negative PrEP users, and sex workers. These data provide insights into transmission between distinct population risk groups, and applied in real-time could allow interventions aimed at controlling gonorrhoea to be provided at a time when current control measures are failing.

Although there was some separation of heterosexual and MSM-associated clusters, this was not absolute, with MSM identified in all major heterosexual-associated clusters, and females identified in six of the seven major MSM-associated clusters. MSMW with gonorrhoea were scattered within MSM and heterosexual networks suggesting a possible role in bridging transmission between MSMO and females, although given our sampling frame, it is not possible to say whether these were responsible for the commencement of transmission clusters. Previous studies (predominantly using NG-MAST) have varied in the extent of bridging identified across heterosexual and MSM-associated networks^23^. For example, Choudhury et al. found that MSM comprised only 1.7% of cases associated with the 14 major heterosexual NG-MAST groups in a large study from London in 2004^24^. In contrast, the proportion of MSM in major heterosexual-associated clusters in our setting ranged from 8% to 24%. This difference may partly reflect the contemporary nature of our study, in the context of changing societal attitudes to reporting of sexual risk factors. Given our observation suggesting bridging transmission, it is possible that measures to reduce gonorrhoea incidence in MSM (including MSMW) may provide an additional benefit of reducing the incidence in heterosexual populations.

Approximately one quarter of HIV-negative MSM in our study reported PrEP use, representing ~16% of all PrEP users in Victoria in 2017^25^. The use of PrEP is highly effective in preventing HIV, and has been associated with a reduction in new HIV diagnoses in Australia^11,26^. However, recent studies, both in the United States and Australia, have demonstrated that uptake of PrEP use in MSM may be accompanied by increased condomless sex, both in PrEP users and in HIV-negative MSM not using PrEP—so-called community-level risk compensation^11,27^. Further, recent epidemiological work in our setting has demonstrated that STIs (gonorrhoea; chlamydia; syphilis) were more common in individuals following receipt of PrEP, and that these STIs were highly concentrated amongst a subset of PrEP users^12^. Here, using genomic analyses, we corroborate these epidemiological findings, and demonstrate that PrEP users are more likely to have repeat infections, or be part of larger transmission clusters. Collectively, these data highlight the need for ongoing, frequent screening of individuals on PrEP.

In our study, all major MSM-associated *N. gonorrhoeae* clusters included HIV-positive and HIV-negative men, providing compelling evidence for sexual mixing and gonococcal transmission between HIV-positive and HIV-negative MSM—including those not taking PrEP. Although it is likely that most HIV-infected patients in our setting are receiving antiretroviral therapy (ART) with undetectable viral load (posing no risk for HIV transmission), our observation of gonorrhoea transmission across HIV risk groups has potential implications for the spread of HIV in other geographic settings where adherence or access to ART is problematic. Moreover, our data further highlight the need for improved access to PrEP in high-risk individuals, such as those in our study with confirmed STI transmission.

Female sex workers with gonorrhoea were scattered in primarily heterosexual networks, suggesting sex workers may have played a role in facilitating transmission. Oropharyngeal gonorrhoea was the most common site of infection in female sex workers (occurring in approximately three-quarters of female sex workers in this study). In Victoria, sex work is legal, although under the Sex Work Act (1994) and Sex Work Regulations (2006), quarterly screening for HIV and other STIs is required. Here, we demonstrate that oropharyngeal gonorrhoea in female sex workers is part of major heterosexual-associated transmission networks, despite previous work in Australia suggesting a high level of condom use during fellatio with male clients^28^. Recently, kissing has been suggested as a risk factor for oropharyngeal gonorrhoea amongst MSM^29^, but to date, there have been no studies investigating kissing as a risk factor for oropharyngeal gonorrhoea in sex workers.

Patients who were likely to have acquired gonorrhoea overseas were more likely to have drug-resistant isolates, and have non-cluster-associated isolates. In addition, travel-associated gonorrhoea was disproportionately higher in heterosexual males, compared to locally acquired cases. The reasons for these observations are unclear, although it is possible that patients who engage in high-risk sexual behaviour overseas are more likely to present for testing and treatment on their return, either because of symptomatic disease (e.g. in heterosexual males returning with urethritis), or concerns about high-risk activities. Here, we provide additional genomics-based evidence for the role of returning travellers in gonorrhoea transmission. Although more than half of travel-associated cases were non-cluster-associated, in ~40% of travel-containing clusters, the earliest isolate in the cluster was from an individual who had likely acquired their gonorrhoea overseas. These data further highlight the importance of providing targeted advice to travellers about the prevention and detection of STIs, which should include testing after sexual contact overseas to ensure effective treatment, and to minimise the potential for subsequent gonorrhoea transmission, including AMR strains.

Of particular concern was the high rate of azithromycin resistance in our setting, identified in 14% of isolates. For comparison, in 2016, the rate of azithromycin resistance in the United States was 3.6%, and in 2017 in England and Wales was 9.2%^30,31^. Approximately 45% of azithromycin-resistant isolates in our setting belonged to a single transmission cluster mainly transmitted between men, where low-level azithromycin resistance was mediated by a mosaic *mtr* locus. This finding is similar to a US study that demonstrated the emergence of azithromycin-resistant *N. gonorrhoeae* isolates from 2014 to 2016 with mosaic *mtr* loci, with the major lineage being MLST 9363^22^. Our contemporary data show that transmission of isolates with interspecies recombination is also the major mechanism of azithromycin resistance in our setting, and demonstrate the intercontinental dissemination of this azithromycin-resistant MLST 9363 lineage, particularly amongst MSM. The apparent rapid global emergence of this lineage is likely to impact on the practicality of developing stable diagnostic molecular tests for azithromycin resistance and highlights the importance of genomic surveillance as a tool for ensuring appropriate diagnostic targets.

Key strengths of our study include our contemporary sampling frame, comprehensive coverage of all cultured cases of gonorrhoea, and integration of individual-level behavioural data. Previous studies of gonorrhoea have demonstrated high interconnectivity of MSM populations in urban Australia^32^, meaning that our findings are likely to have applicability across major cities in Australia. A limitation of this work was the relatively low proportion of isolates included from females (13%) and heterosexual males (12%) compared to MSM (75%). It is likely that the lower proportion of *N. gonorrhoeae* isolates from females reflects the overall epidemiology of gonorrhoea in our setting—in 2017, females represented only 18.6% of all gonorrhoea notifications (i.e. culture plus NAAT) in Victoria^7^. This caveat is not unique to our study—for example, in a previous study assessing gonorrhoea transmission in London and Brighton in the UK, females comprised only 6% of the total dataset^18^. Similarly, in a recent WGS-based European study, only 15% of isolates were from females^15^. Further, ~10% of isolates in our study were from males with unknown sexual risk factors; it is possible that a proportion of these isolates were from heterosexual males. Future work should attempt to increase the number of cultures obtained from females and heterosexual males to provide additional validation of our observations. Although we received all cultured isolates in the state during the study, this represented only 28% of all gonorrhoea notifications over the study period. Again, this limitation is not unique to our study, and applies to all WGS-based studies of gonorrhoea, whereby the increasing use of molecular testing reduces the availability of isolates for additional analyses. In an era of increasing AMR in STIs, it is critical that concerted efforts are made to ensure continuation of culture-based surveillance, particularly in light of emerging resistance mechanisms^21^.

In conclusion, we demonstrate transmission of gonococcal lineages within and across distinct sexual networks and identify several potential touchpoints that may promote the dissemination of *N. gonorrhoeae*, namely PrEP use, oropharyngeal gonorrhoea in female sex workers, returning international travellers, and MSMW who may facilitate bridging between MSMO and heterosexuals. Specific public health findings of our study include the need for ongoing STI screening (particularly amongst high-risk groups, such as PrEP users, sex workers, and returning travellers); transmission of gonorrhoea amongst HIV serodiscordant individuals (posing a theoretical ongoing risk for HIV transmission in individuals not on PrEP); the intercontinental emergence of an azithromycin-resistant lineage (further highlighting the role of mosaicism in *N. gonorrhoeae* resistance development, and demonstrating the need for genomic surveillance in detecting the global emergence of such strains), and bridging of *N. gonorrhoeae* between MSM and heterosexuals (highlighting the utility of genomics in defining the broader risk networks for gonorrhoea transmission). The methods we have employed in this study, translated into real-time clinical practice, could facilitate rapid and precise interventions for gonorrhoea control. The resolution afforded by the integration of detailed patient-level data with WGS demonstrates the complex epidemiological and behavioural factors that drive *N. gonorrhoeae* transmission and highlights the necessity for equally sophisticated approaches to prevention and control.

## Methods

### Setting, patients and data sources

In Australia, all gonorrhoea cases are notified to public health authorities in each State or Territory. Diagnostic laboratories are requested to forward *N. gonorrhoeae* isolates to a reference laboratory for further characterisation. The Microbiological Diagnostic Unit Public Health Laboratory (MDU PHL) is the sole public health bacteriology reference laboratory for the State of Victoria in Australia, covering a resident population of ~6.24 million. All cultured *N. gonorrhoeae* isolates in Victoria (from sexual health clinics and primary care services) are forwarded to MDU PHL for antimicrobial susceptibility testing (AST) (see below).

We conducted a retrospective, observational study of all patients in Victoria with clinical cultures positive for *N. gonorrhoeae* between January 1, 2017 and December 31, 2017. Samples included those collected from asymptomatic and symptomatic adult patients (>16 years), and from genital and extra-genital (oropharyngeal and rectal) sites. Detailed demographic and sexual behavioural information on patients was obtained from case report forms from the Victorian Department of Health and Human Services, or from computer-assisted self-interview records from Melbourne Sexual Health Centre (MSHC), the major publicly funded sexual health centre in Victoria. Data collected included gender, age, site of infection, sexual risk group (e.g. MSM, heterosexual males or females), HIV status, current use of HIV pre-exposure prophylaxis (PrEP), and whether the patient was a sex worker. Where relevant, we subclassified MSM risk behaviour into men who have sex with men only (MSMO) and men who have sex with men and women (MSMW). Information on gonorrhoea notifications in Victoria was obtained from the National Notifiable Diseases Surveillance System (NNDSS)^7^.

### Microbiological testing

All microbiological testing was performed at MDU PHL. Single colonies of presumptive *N. gonorrhoeae* were selected from the primary culture plate for speciation, AST and subsequent DNA extraction. Isolates were confirmed as *N. gonorrhoeae* on the MALDI Biotyper (Bruker Daltonik, Bremen, Germany). AST was performed in accordance with the Australian Gonococcal Surveillance Programme (AGSP) using agar breakpoint dilution on GC agar for the following antimicrobials: azithromycin, ceftriaxone, ciprofloxacin, penicillin, spectinomycin, and tetracycline^33^. Resistance to antimicrobial agents was defined as in Supplementary Table 5.

### DNA extraction and WGS

DNA extraction and WGS of study isolates was performed at MDU PHL. Genomic DNA was extracted from a single colony using a QIAsymphony™ DSP DNA Mini Kit (Qiagen) according to manufacturer’s instructions, and WGS was performed on an Illumina NextSeq 500 instrument with 150 bp paired-end reads using Illumina libraries and protocols (Illumina, San Diego, CA, USA).

### Phylogenetic analysis and cluster generation

Reads were trimmed to remove adaptor sequences and low-quality bases (*Q* < 10) with Trimmomatic v0.38^34^. Kraken (v2.0.7) was used to investigate for contamination^35^. The 2186*Neisseria gonorrhoeae* genomes were aligned to the NCCP11945 reference genome (GenBank accession NC_011035.1) using Snippy v4.3.5 (https://github.com/tseemann/snippy). Alignment was performed using BWA MEM v0.7.17^36^, and single nucleotide polymorphisms (SNPs) were called using Freebayes v1.2.0, requiring a minimum read coverage of 10, minimum base quality of 13% and 90% read concordance at a site for a SNP to be reported. The resulting core SNP alignment consisted of 41,145 sites. Maximum likelihood (ML) phylogenetic trees were inferred using IQ-tree (v1.6.8)^37^, with the best-fitting nucleotide substitution model chosen based on the lowest Bayesian Information Criterion. Trees were loaded into R and visualised alongside metadata using *ggtree*^38^. The core SNP alignment was loaded into R, and pairwise SNP distances were calculated using the dist.dna function in *ape*^39^. Hierarchical single-linkage cluster analysis was performed using the hclust function in the R package *stats*, and clusters were filtered using a threshold of 10 SNPS using the cutree function in *stats*.

### Phylogeographic analysis of MLST 9363 isolates

For phylogeographic context, we performed additional phylogenetic analysis using 151 MLST 9363 isolates from a recent study in the United States (Supplementary Data 2)^21^, and 388 MLST 9363 isolates from this study. To maximise comparability with previously described analyses of MLST 9363, we performed phylogenetic analysis as closely as possible to that described by Thomas et al.^22^. Briefly, read-mapping was performed as above, with recombination removed using Gubbins^40^. The resulting core SNP alignment consisted of 1730 sites. ML phylogenetic trees were inferred using IQ-tree (v1.6.8)^37^, with the best-fitting nucleotide substitution model chosen based on the lowest Bayesian Information Criterion. Trees were loaded into R and visualised alongside metadata using *ggtree*^38^. Clustering was performed on the recombination-filtered alignment using RAMI^41^, with a patristic distance threshold of 0.000005.

### Genome assemblies and detection of resistance determinants

De novo assembly was performed using SPAdes (v.3.12.1)^42^, and genes were annotated using Prokka^43^. v1.13.43 NG‐MAST STs were assigned using NGmaster v0.5.5^44^, with data from the NG‐MAST database (http://www.ng-mast.net). MLST STs were identified using MLST v2.15 (https://github.com/tseemann/mlst) with the PubMLST database (http://pubmlst.org/neisseria). ARIBA v2.13 was used to identify known antimicrobial resistance determinants and variants in *N. gonorrhoeae*^15,45^. Additional mutations in *penA* and *penB*, and in the *mtr* operon were identified in de novo assemblies using BLAST+ v2.7, and manually inspected in Geneious (v11.1.13). Mosaic *mtr* loci were compared for nucleotide sequence similarity using BLAST to the FA1090 reference genome (Genbank Accession NC_002946.2). Similar mosaic *mtr* loci to those identified in Australian isolates were identified in international datasets by downloading sequence data from the United States (BioProject PRJNA317462) and the United Kingdom (BioProject PRJNA315363)^18^ and a BLAST search of de novo assemblies (assembled using SPAdes^42^). Comparisons were visualised in R with *genoplotR* v0.8.7^46^. Genotypic AMR determinants were correlated with phenotypic susceptibility data.

### Transmission analysis

To determine the intra-patient genomic diversity of *N. gonorrhoeae*, isolates from patients with infections at multiple sites underwent WGS. In addition, isolates from known sexual partners attending MSHC on the same day were also sequenced to determine the range of SNPs from epidemiologically likely transmission events. This approach was based on a previous study in our setting by Kwong et al. that assessed intra-couple SNP diversity by comparing *N. gonorrhoeae* isolates from 34 male couples in a known sexual partnership^20^.

### Statistical analysis

Associations between isolates or clusters and epidemiological characteristics were made using a chi-squared test. The Mann–Whitney Rank sum test was used to compare non-normal distributions. All statistical analyses were performed using R (version 3.4.0).

### Ethical statement

No individual patient consent was required or sought as data were collected in accordance with the Victorian Public Health and Wellbeing Act 2008^47^. Ethical approval was obtained from the Alfred Hospital Ethics Committee (Project 625/17).

### Reporting summary

Further information on research design is available in the Nature Research Reporting Summary linked to this article.

## Supplementary information

Supplementary Information

Peer Review File

Reporting Summary

Description of Additional Supplementary Files

Supplementary Data 1

Supplementary Data 2

## Data Availability

All sequencing data for isolates in this study are available from the NCBI Sequence Read Archive (BioProject PRJNA520805). Data supporting the findings of this study are available within the text and in Supplementary Information files, or are available from the authors upon request.
