# Supplementary material for: Bridging of Neisseria gonorrhoeae lineages across sexual networks in the HIV pre-exposure prophylaxis era
Source: Nat Commun. 2019 Sep 5;10:3988. doi: 10.1038/s41467-019-12053-4 (PMC6728426; doi:10.1038/s41467-019-12053-4)
Supplement: Supplementary file 1 — Supplementary Information [file 41467_2019_12053_MOESM1_ESM.pdf]

Bridging of *Neisseria gonorrhoeae* lineages across sexual networks in the HIV pre-exposure prophylaxis era

Williamson et al.

SUPPLEMENTARY INFORMATION

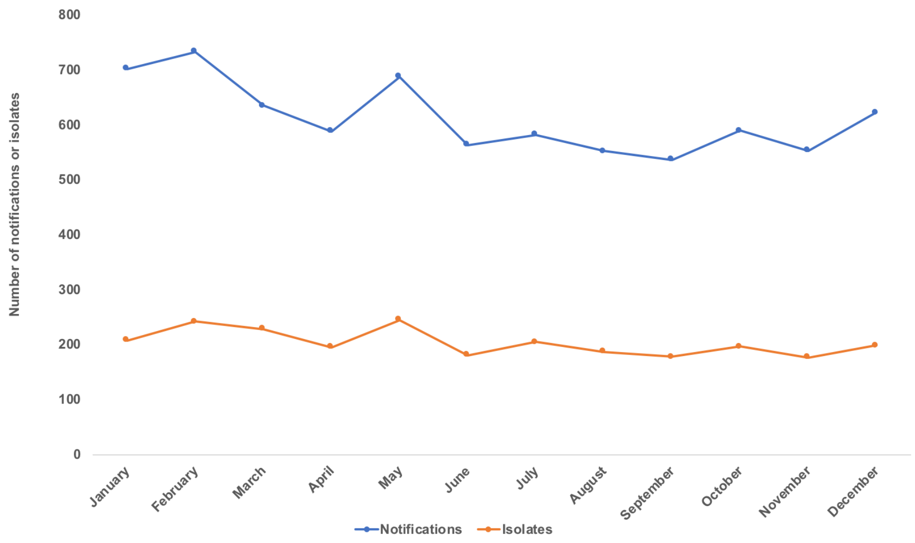

Supplementary Figure 1. Number of gonorrhoea notifications in Victoria received through the National Notifiable Diseases Surveillance System in 2017 (notifications) compared to the number of *Neisseria gonorrhoeae* isolates cultured at the Microbiological Diagnostic Unit Public Health Laboratory, 2017 (isolates).

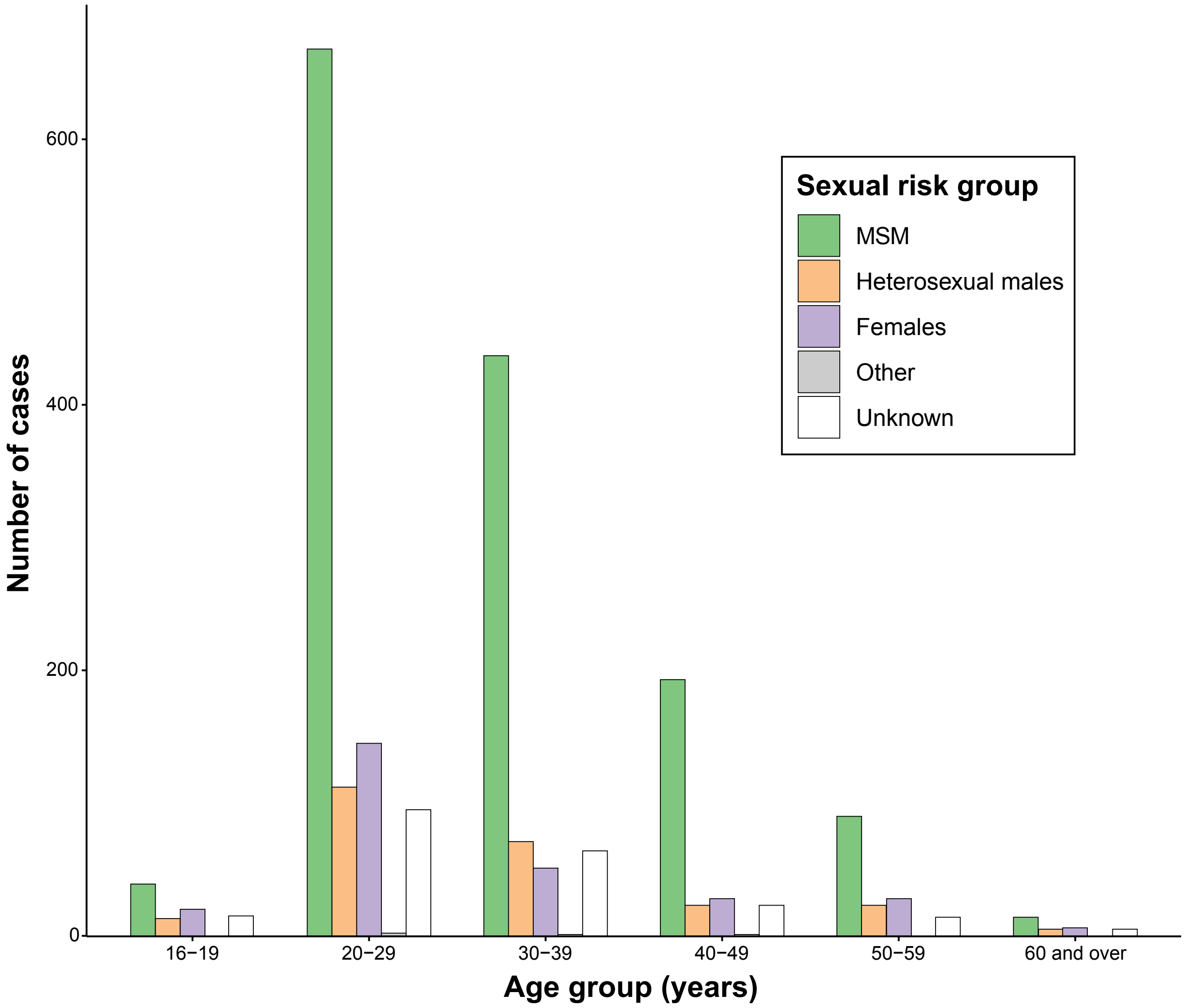

Supplementary Figure 2. Age, sex and sexual risk group distribution of cases included in this study.

A.

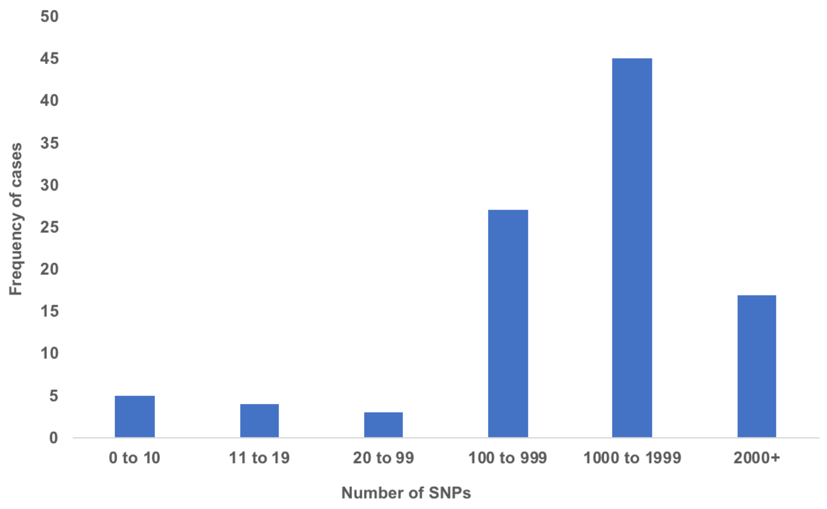

B.

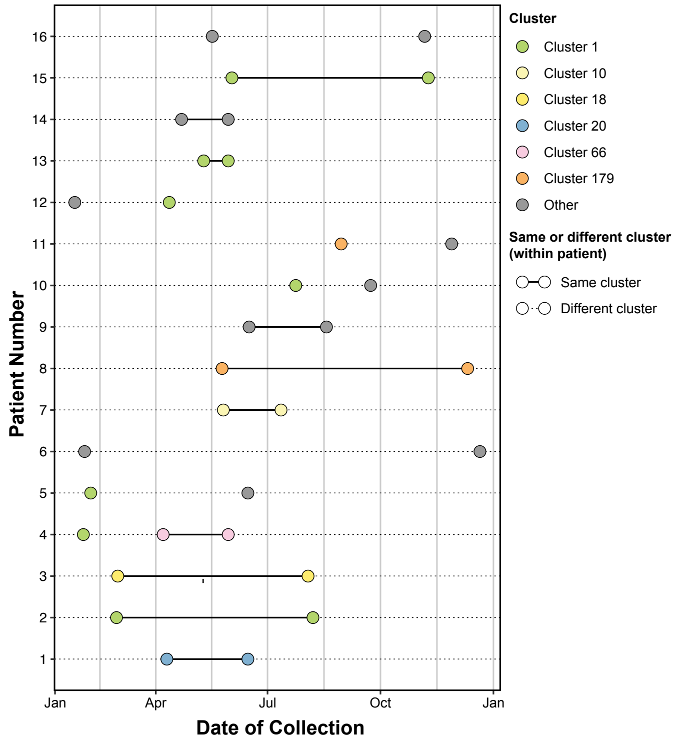

Supplementary Figure 3. (A) Number of SNPs between first and last *Neisseria gonorrhoeae* isolates in patients with two or more isolates taken ≥14 days apart. 88 patients had two or more isolates separated by ≥14 days, with >100 SNPs between isolates. (B) Characteristics of sixteen patients who had two or more isolates separated by ≥14 days, with <100 SNPs between isolates. Nine patients had repeat isolates that belonged to the same transmission cluster.

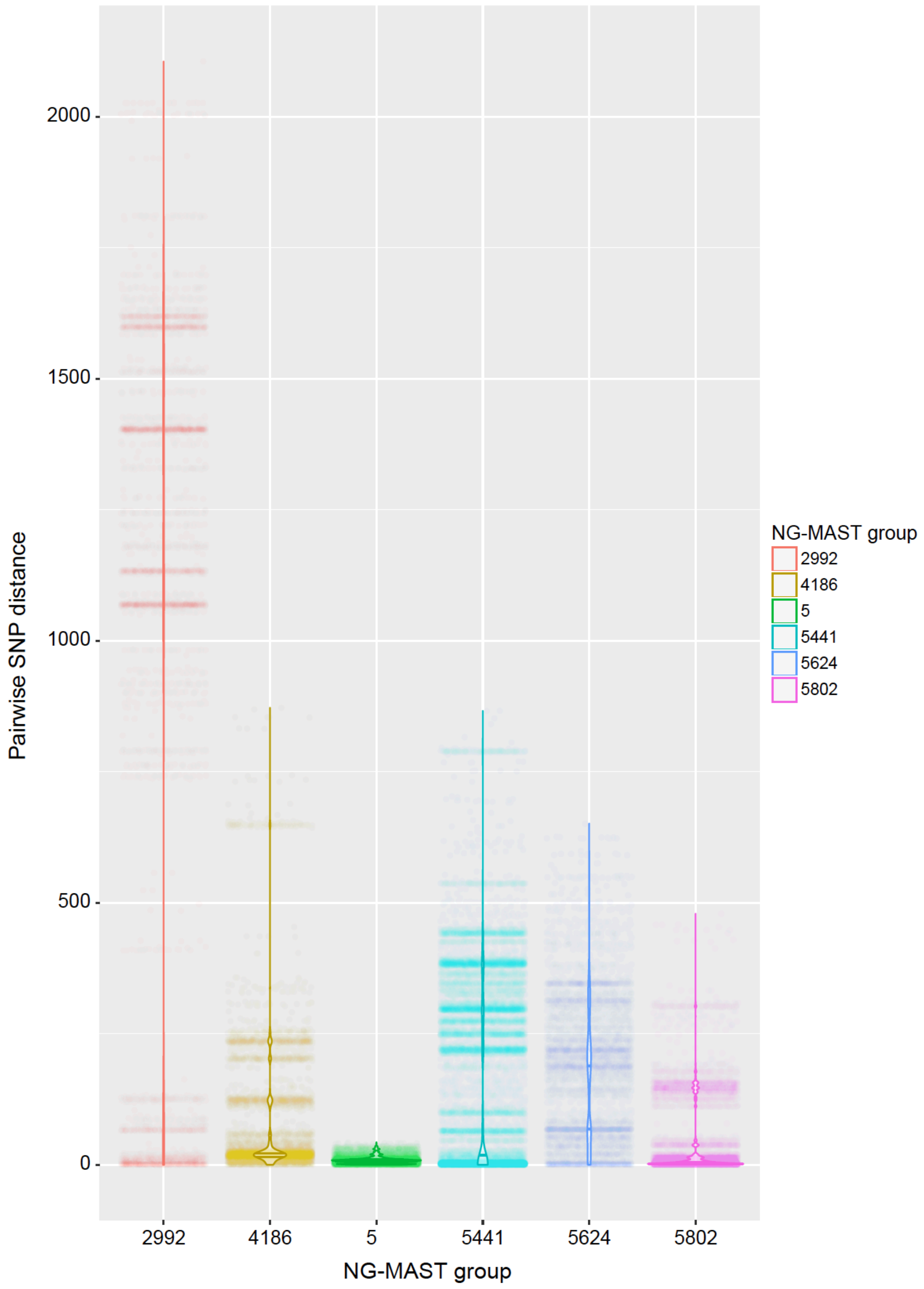

Supplementary Figure 4. Violin plots showing pairwise single nucleotide polymorphism (SNP) distributions of isolates in each of the six major NG-MAST groups.

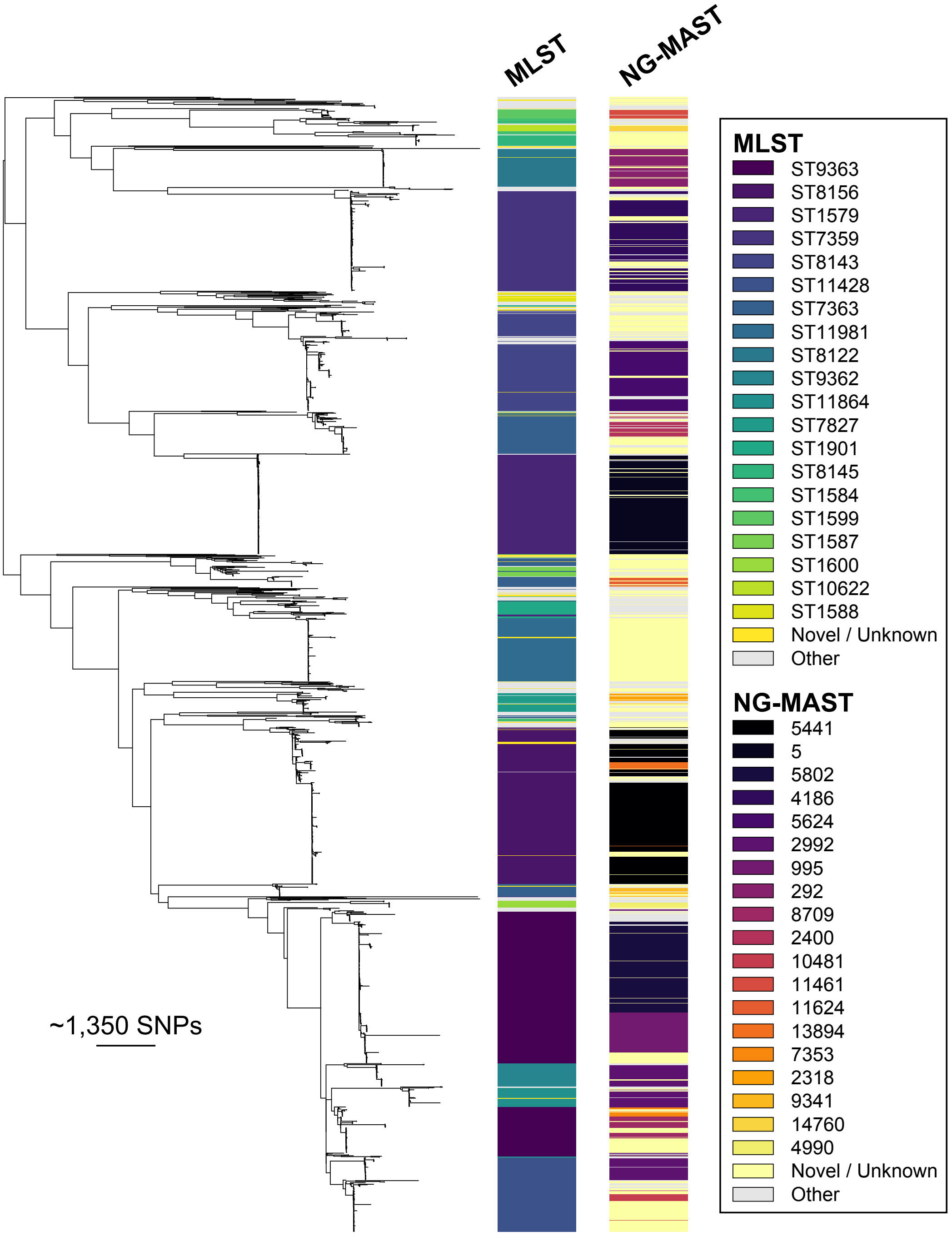

Supplementary Figure 5. Population structure of 2,186 *N. gonorrhoeae* isolates included in this study. The mid-point rooted maximum-likelihood tree (derived from 45,145 core SNPs) is plotted on the left. The NG-MAST and MLST profiles are shown on the right. The scale bar represents single nucleotide polymorphisms (SNPs).

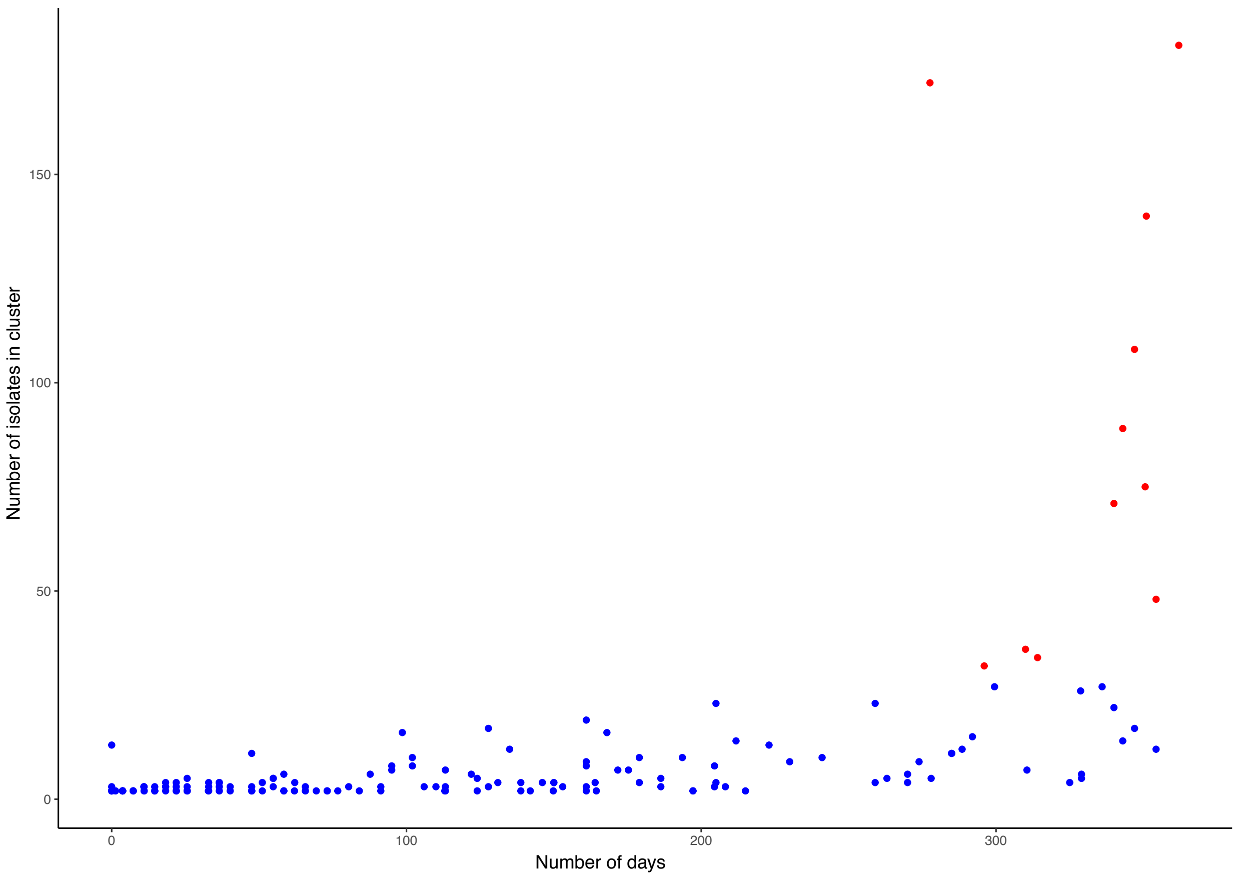

Supplementary Figure 6. Duration and size of genetic clusters of *Neisseria gonorrhoeae* isolates. Each point represents a cluster, with red points indicating the eleven major clusters (each comprising ≥30 patients) investigated further in this study.

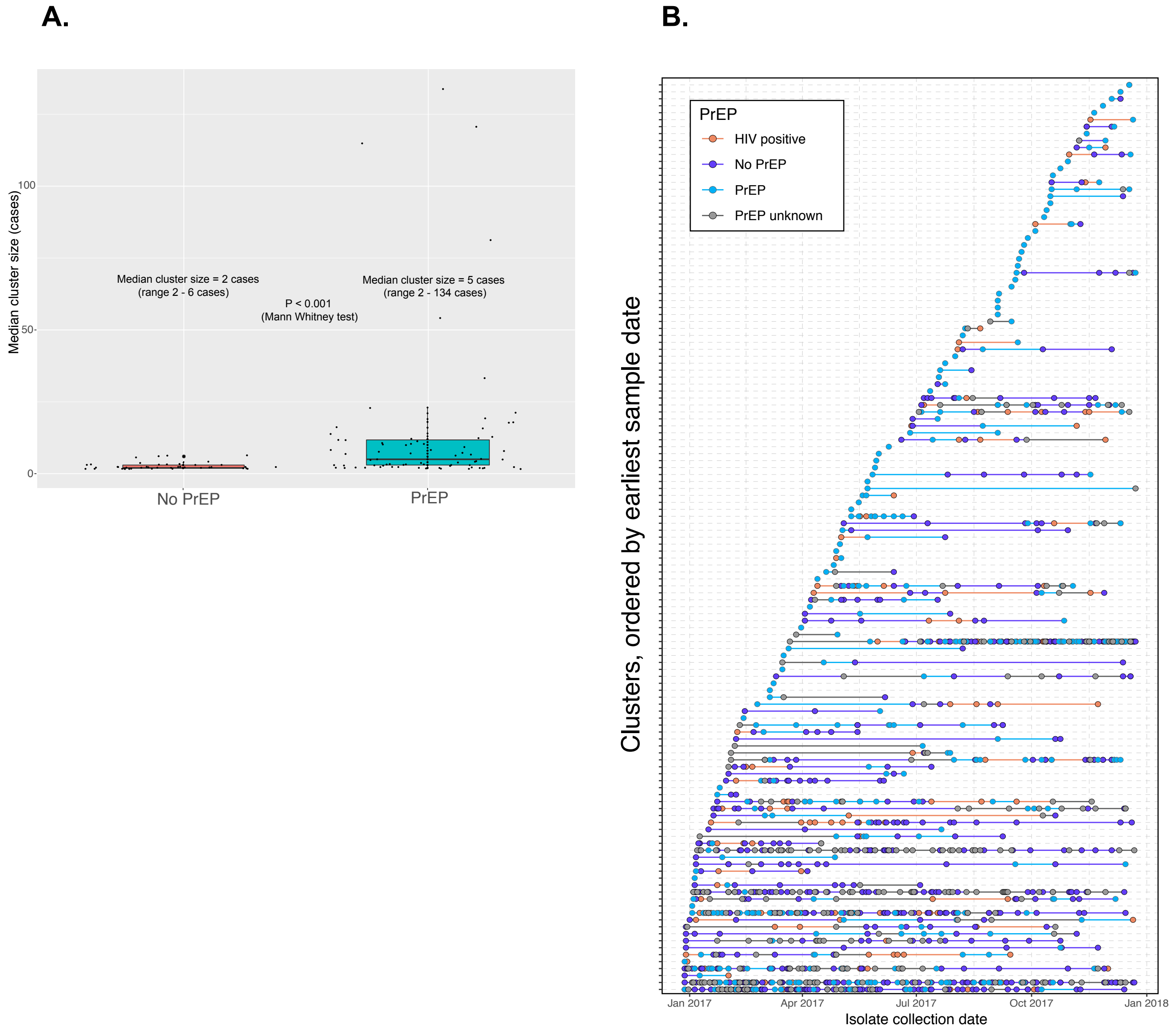

Supplementary Figure 7. Characteristics of clusters containing individuals receiving HIV pre-exposure prophylaxis (PrEP). (A) Median size of clusters containing HIV-negative MSM receiving PrEP and HIV-negative MSM not receiving PrEP. Box plots indicate median and interquartile range (IQR), with the whiskers representing the highest and lowest values within 1.5×IQR of the upper and lower quartiles. (B) Timeline of *Neisseria gonorrhoeae* clusters stratified by HIV status and PrEP use. The x-axis represents time (based on data of sample collection), and each dot within the plots represents a single isolate. Data points are coloured by risk factor.

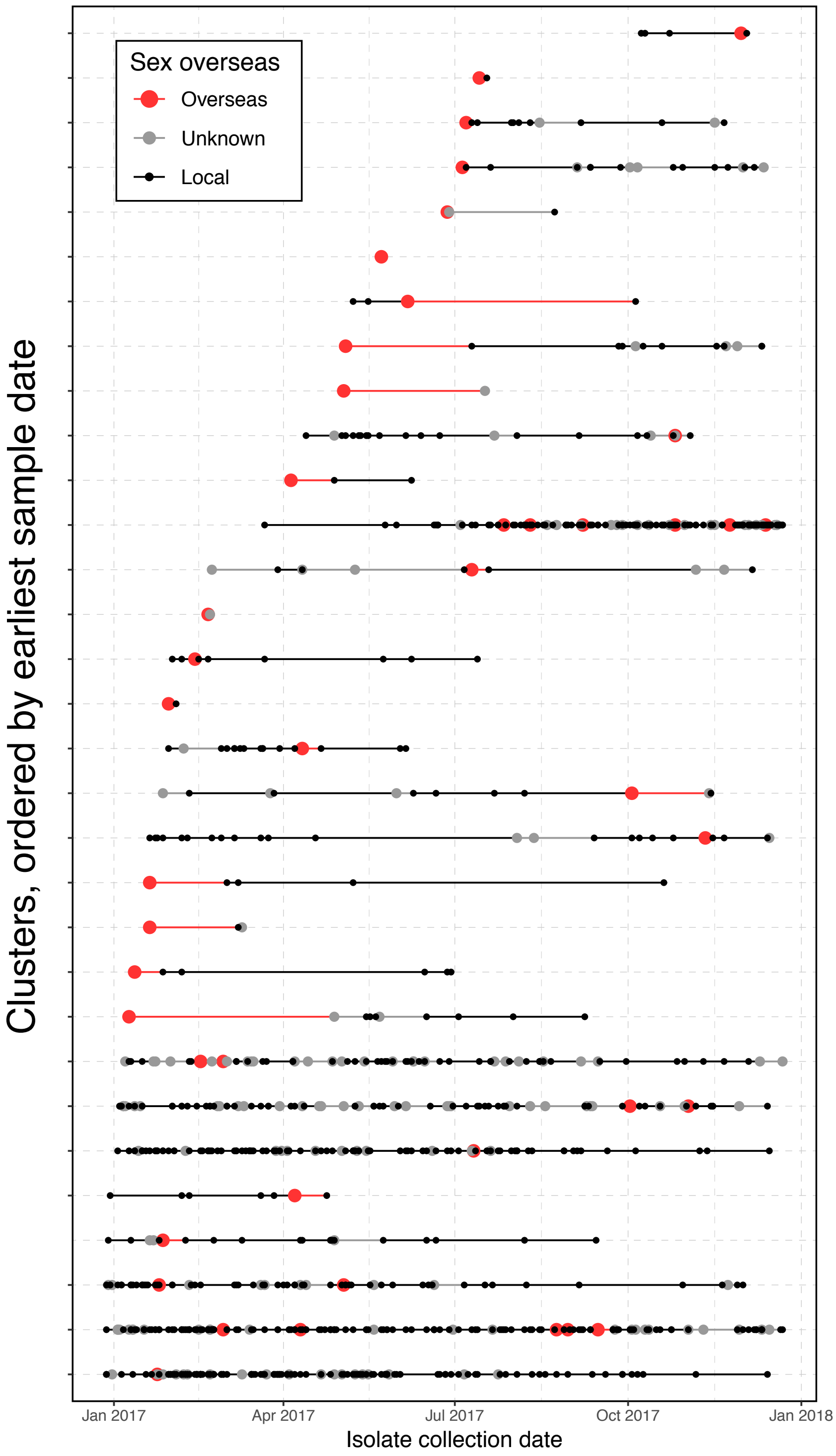

Supplementary Figure 8. Timeline of *Neisseria gonorrhoeae* clusters, stratified by overseas travel. The x-axis represents time (based on data of sample collection), and each dot within the plots represents a single isolate. Data points are coloured by risk factor.

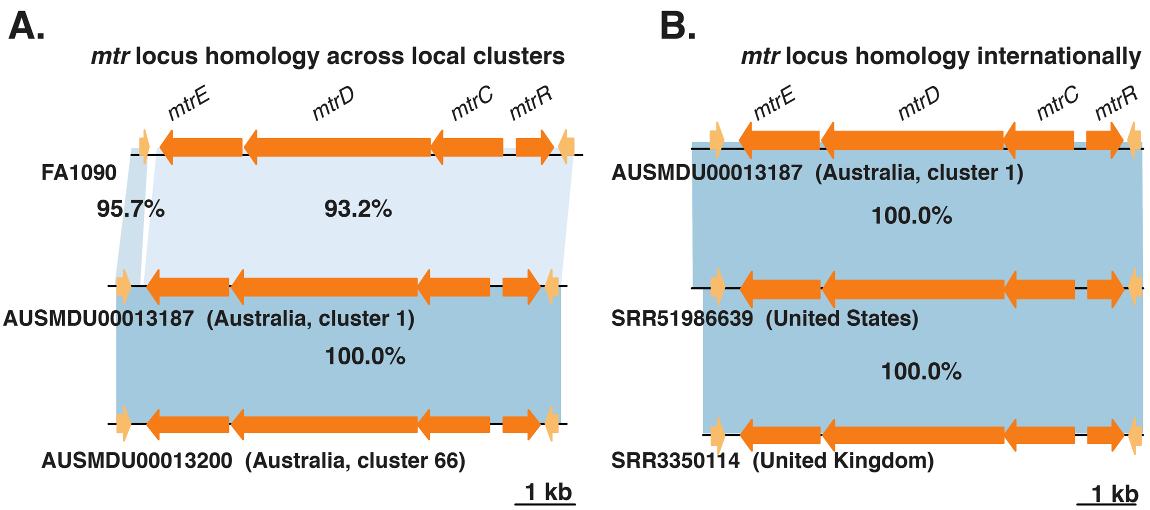

Supplementary Figure 9. Schematic of wild-type and *Neisseria meningitidis*-like mosaic *mtr* loci. Comparison of *mtr* loci associated with low-level azithromycin resistance in Australian *Neisseria gonorrhoeae* isolates with (A) FA 1090 *N. gonorrhoeae* reference genome (Genbank Accession NC_002946.2) and (B) Two publicly available *N. gonorrhoeae* sequences containing identical mosaic *mtr* loci. Isolates SRR5198639 and SRR3350114 are available at NCBI (BioProjects PRJNA317462 and PRJNA315363, respectively).

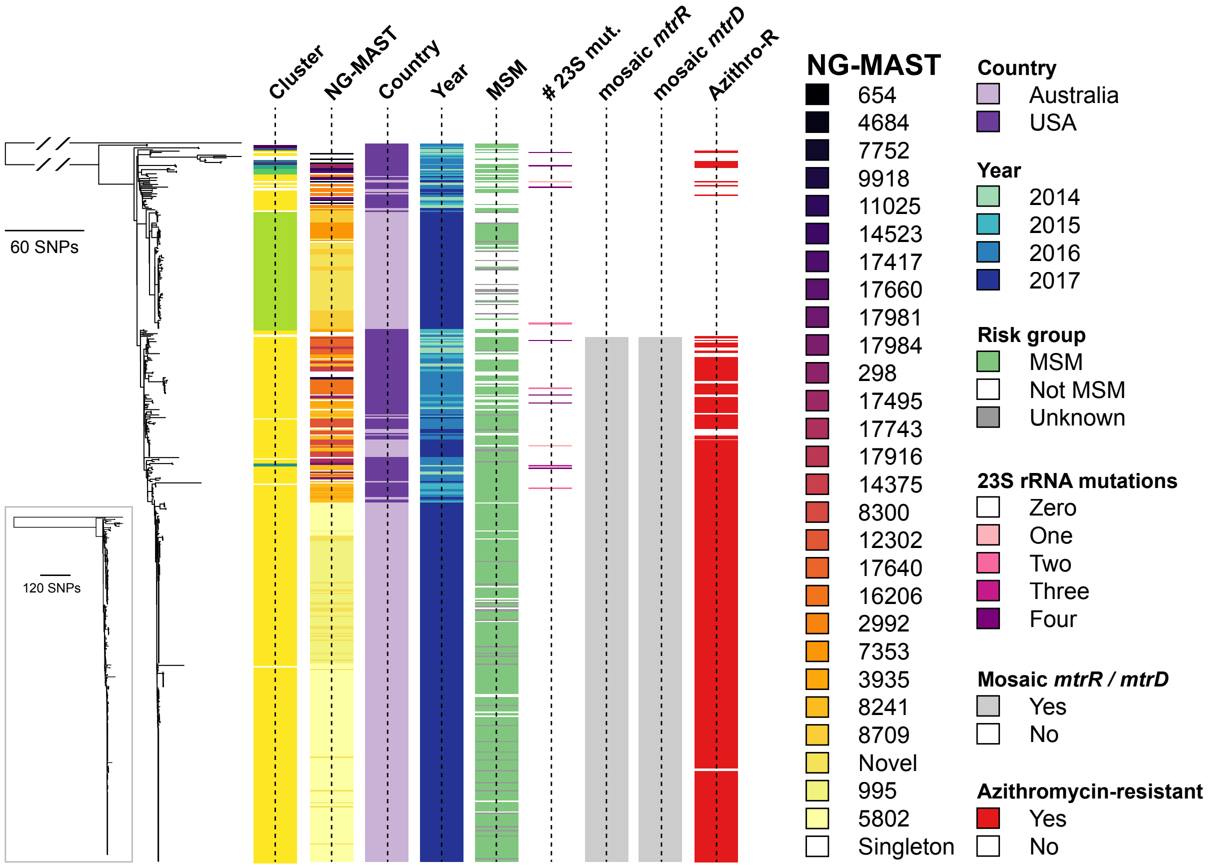

Supplementary Figure 10. Australian *Neisseria gonorrhoeae* multilocus sequence type (MLST) ST9363 isolates in the context of 151 recently described MLST 9363 isolates from the United States. The heatmap displays: (i) RAMI cluster; (ii) NG-MAST type; (iii) Country of isolation; (iv) Year of isolation; (v) Whether the isolate was from a case reporting male-to-male sexual contact; (vi) Number of 23S rRNA alleles with mutations, relative to the FA1090 reference and (vii) Presence of mosaic *mtrR* allele described by Thomas et al.[1] (vii) Presence of *Neisseria meningitidis*-like mosaic *mtrD* allele described by Thomas et al.,[1] and (viii) Reported phenotypic azithromycin resistance. The scale bars represents single nucleotide polymorphisms (SNPs).

Supplementary Table 1. Characteristics of patients with two or more gonorrhoea infections in this study.

Characteristic [a]	One infection	≥2 infections [b]	*P*
Male	1,675/1,951 (85.8)	88/88 (100)	<0.01
MSM	1,229/1,741 (70.6)	87/88 (97)	<0.01
HIV-positive	199/1,638 (12.1)	19/88 (21.6)	<0.01
PrEP use [c]	286/1,290 (22.2)	28/66 (42.4)	<0.01
Reported sex work	76/1,556 (4.9)	3/81 (3.7)	0.79
Median age	29.9 (25.2-37.2)	29.3 (25.1-36.3)	0.86

Abbreviations: MSM men who have sex with men; PrEP, pre-exposure prophylaxis

[a] Denominator based on whether risk factor data for each characteristic reported
[b] >100 SNPs between isolates collected ≥14 days apart
[c] Excluding HIV-positive patients

Supplementary Table 2. Characteristics of patients in the eleven most common *Neisseria gonorrhoeae* lineages, Victoria, Australia, 2017

**Characteristic**	**Genetic cluster (number in cluster)**											***P***
	**10 (181)**	**179 (172)**	**1 (140)**	**20 (108)**	**24 (89)**	**32 (75)**	**5 (71)**	**18 (48)**	**94 (36)**	**91 (34)**	**66 (32)**	
**Median age (years, IQR)**	29.1 (24.9-34.8)	36.4 (28.4-45.8)	28.4 (23.0-32.9)	32.5 (27.6-41.5)	28.6 (23.9-34.7)	29.6 (25.3-37.0)	30.3 (26.5-30.7)	27.5 (24.3-35.4)	29.3 (24.7-35.0)	27.5 (22.2-32.3)	34.4 (29.4-42.7)	<0.01[e]
**Males (%)**	160 (88.4)	169 (98.3)	135 (96.4)	99 (91.7)	46 (51.7)	58 (77.3)	70 (98.6)	31 (64.6)	36 (100)	14 (41.2)	30 (93.8)	<0.01[f]
**MSMO (%)** [a]	131/168 (78.0)	149/162 (92.0)	115/128 (89.8)	84/100 (84)	5/74 (6.8)	12/51 (23.5)	56/63 (88.9)	1/38 (2.6)	35/35 (100)	3/32 (9.4)	25/29 (86.2)	<0.01[f]
**MSMW (%)** [a]	3/168 (1.8)	8/162 (4.9)	6/128 (4.7)	2/100 (2)	1/74 (1)	0	0	2/38 (5.3)	0	0	1/29 (3.4)	ND
**Heterosexuals (%)** [a]	34/168 [13M;21F] (20.2)	5/162 [2M;3F] (8.9)	7/128 [2M; 5F] (5.5)	14/100 [7M; 7F] (14)	68/74 [27M;41F] (91.9)	39/51 [22M;17F] (76.5)	7/63 [6M; 1F] (11.1)	35/38 [18M;17F] (92.1)	0/35 (0)	29/32 [9M; 20F] (90.6)	3/29 [1M; 2F] (10.3)	<0.01[f]
**Sex work (%)** [b]	10/153 [3M; 2F] (6.5)	3/153 [2M; 1F] (2.0)	4/121 [3M; 1F] (3.3)	3/90 [2M; 1O] (3.3)	7/59 [5F; 2O] (11.9)	5/40 [5F] (12.5)	4/57 [4M] (7.0)	1/26 [1F] (3.8)	1/33 [1M] (3.0)	6/24 [5F; 1M] (16.7)	2/27 [2M] (7.4)	<0.01[f]
**HIV positive (%)** [c]	13/162 (8.0)	44/162 (27.2)	7/127 (5.5)	15/100 (15)	0/60 (0)	1/44 (2.3)	7/62 (11.3)	0/28 (0)	3/34 (8.8)	0/26 (0)	8/28 (28.6)	<0.01[f]
**PrEP (%)** [d]	40/135 (29.6)	42/110 (38.2)	24/114 (21.1)	27/76 (35.5)	1/47 (2.1)	2/35 (5.7)	18/49 (36.7)	0/21 (0)	12/29 (41.4)	0/20 (0)	10/18 (55.6)	<0.01[f]

Abbreviations: IQR, interquartile range; MSMO, men who have sex with men only; MSMW, men who have sex with men and women; PrEP, pre-exposure prophylaxis; M, male; F, female; ND, not determined as numbers too small. Shaded rows represent lineages with >70% MSM

[a] Denominator value is where risk behaviour was reported

[b] Denominator value is where information on current sex work was reported

[c] Denominator value is where HIV status was reported

[d] Denominator value is for HIV negative patients where PrEP use was reported

[e] Kruskal-Wallis one-way analysis of variance test

[f] As determined by χ^2 test

Supplementary Table 3: Characteristics of gonorrhoea infections from patients reporting sex overseas compared to patients with locally-acquired infections.

**Characteristic**	**Isolates from patients reporting sex overseas**	**Isolates from patients reporting local acquisition**	***P***
**Median age, years (IQR)** [a]			
All individuals	30.2 (24.7-38.4)	29.8 (25.2-37.1)	0.98
MSM	30.2 (24.5-35.0)	30.2 (25.9-37.5)	0.10
Females	24.1 (24.1-38.6)	26.4 (22.9-36.4)	0.78
Heterosexual males	32.7 (27.6-51.3)	29.8 (24.7-36.0)	0.03
**Number of isolates as part of a cluster**	44/96 (45.8)	1,387/1,616 (85.8)	<0.01
**Sexual risk** [b]			
MSM	52/96 (54.2)	1,203/1,616 (74.4)	<0.01
Females	11/96 (11.5)	183/1,616 (11.3)	0.99
Heterosexual males	31/96 (32.3)	192/1,616 (11.9)	<0.01
Unknown	2/96 (2.1)	38/1,616 (2.4)	0.99
**Antimicrobial resistance**	**Number resistant / Number tested (% resistant)**		
Penicillin [c]	40/96 (41.7)	479/1,616 (29.6)	0.01
Tetracycline [d]	29/96 (30.2)	91/1,616 (5.6)	<0.01
Ceftriaxone [e]	1/96 (1)	36/1,616 (2.2)	0.48
Ciprofloxacin	50/96 (52)	492/1,616 (30.4)	<0.01
Azithromycin	5/96 (5.2)	230/1,616 (14.2)	0.01

Abbreviations: IQR, interquartile range; MSM, men who have sex with men

[a] Median age of bisexual males and males with unknown sexual risk factor not assessed due to small number of cases

[b] Denominator value is where sexual orientation was reported

[c] All penicillinase-producing *Neisseria gonorrhoeae* (PPNG) isolates are considered resistant to penicillin, irrespective of the MIC value

[d] The Australian Gonococcal Surveillance Program (AGSP) monitors for high-level resistance to tetracycline (TRNG) only. As such, TRNG is used for epidemiological surveillance purposes only.[2]

[e] Decreased susceptibility

Supplementary Table 4: Antimicrobial susceptibility profile of *Neisseria gonorrhoeae* isolates between 1st January 2017 and 31st December 2017, Victoria, Australia

**Antimicrobial**	**All isolates**	**Isolates from MSM**	**Isolates from females**	**Isolates from heterosexual males**	***P***
	**Number resistant / Number tested (% resistant)**				
**Penicillin** [a]	645/2186 (29.5)	433/1,445 (30.0)	84/278 (30.2)	69/247 (27.9)	0.80
**Tetracycline** [b]	152/2186 (6.9)	82/1,445 (5.7)	23/278 (8.3)	34/247 (13.8)	<0.01
**Ceftriaxone** [c]	46/2186 (2.1)	27/1,445 (1.9)	9/278 (3.2)	6/247 (2.4)	0.33
**Ciprofloxacin**	673/2186 (30.8)	430/1,445 (29.8)	93/278 (33.5)	91/247 (36.8)	0.21
**Azithromycin** [d]	297/2186 (13.7)	246/1,445 (17.0)	15/278 (5.4)	9/247 (3.6)	<0.01

Abbreviations: MSM, men who have sex with men

[a] All penicillinase-producing *Neisseria gonorrhoeae* (PPNG) isolates are considered resistant to penicillin, irrespective of the MIC value

[b] The Australian Gonococcal Surveillance Program (AGSP) monitors for high level resistance to tetracycline (TRNG) only. Tetracyclines are rarely, if ever used for treatment of gonorrhoea in Australia. As such, TRNG is used for epidemiological surveillance purposes only.[2]

[c] Decreased susceptibility

[d] Two high-level azithromycin-resistant (MIC > 256mg/L) isolates were identified from an epidemiologically linked heterosexual couple. Both isolates had A2045G mutations in all four copies of the 23S rRNA alleles, and did not have a mosaic *mtr* locus (Supplementary Dataset 1).

Supplementary Table 5. Australian Gonococcal Surveillance Program (AGSP) interpretative criteria for *Neisseria gonorrhoeae* Minimum Inhibitory Concentration (MIC) values

Antimicrobial	MIC (mg/L)		
	**Sensitive**	**Less susceptible / decreased susceptibility**	**Resistant**
Ceftriaxone	≤ 0.03	0.06–0.125	Not defined
Azithromycin	≤ 0.5	Not defined	≥1
Penicillin [a]	≤ 0.03	0.06–0.5	≥1
Ciprofloxacin	≤ 0.03	0.06–0.5	≥1
Spectinomycin	≤64	Not defined	≥128
Tetracycline [b]	≤ 8	Not defined	≥16

Abbreviations: MIC, minimum inhibitory concentration

[a] All penicillinase-producing *N. gonorrhoeae* (PPNG) isolates are considered resistant to penicillin, irrespective of the MIC value.

[b] The Australian Gonococcal Surveillance Program (AGSP) monitors for high level resistance to tetracycline (TRNG) only. Tetracyclines are rarely, if ever used for treatment of gonorrhoea in Australia. As such, TRNG is used for epidemiological surveillance purposes only.[2]

REFERENCES

1 Thomas, J. C. *et al.* Evidence of Recent Genomic Evolution in Gonococcal Strains with Decreased Susceptibility to Cephalosporins or Azithromycin in the United States, 2014-2016. *J Infect Dis* 220(2): 294-305 (2019).

2 Lahra, M. M., Enriquez, R. & George, C. R. R. Australian Gonococcal Surveillance Programme Annual Report, 2017. *Commun Dis Intell* 15;43 (2019).
