# Supplementary material for: Bridging of Neisseria gonorrhoeae lineages across sexual networks in the HIV pre-exposure prophylaxis era
Source: Nat Commun. 2019 Sep 5;10:3988. doi: 10.1038/s41467-019-12053-4 (PMC6728426; doi:10.1038/s41467-019-12053-4)
Supplement: Supplementary file 3 — Reporting Summary [file 41467_2019_12053_MOESM3_ESM.pdf]

nature research

Corresponding author(s): *Double-blind peer review submissions: write DBPR and your manuscript number here instead of author names.*

Last updated by author(s): *YYYY-MM-DD*

Reporting Summary

Nature Research wishes to improve the reproducibility of the work that we publish. This form provides structure for consistency and transparency in reporting. For further information on Nature Research policies, see Authors & Referees and the Editorial Policy Checklist .

Statistics

For all statistical analyses, confirm that the following items are present in the figure legend, table legend, main text, or Methods section.

n/a	Confirmed	
☐	☒	The exact sample size (n) for each experimental group/condition, given as a discrete number and unit of measurement
☐	☒	A statement on whether measurements were taken from distinct samples or whether the same sample was measured repeatedly
☐	☒	The statistical test(s) used AND whether they are one- or two-sided<br>*Only common tests should be described solely by name; describe more complex techniques in the Methods section.*
☒	☐	A description of all covariates tested
☒	☐	A description of any assumptions or corrections, such as tests of normality and adjustment for multiple comparisons
☐	☒	A full description of the statistical parameters including central tendency (e.g. means) or other basic estimates (e.g. regression coefficient) AND variation (e.g. standard deviation) or associated estimates of uncertainty (e.g. confidence intervals)
☒	☐	For null hypothesis testing, the test statistic (e.g. F, t, r) with confidence intervals, effect sizes, degrees of freedom and P value noted<br>*Give P values as exact values whenever suitable.*
☒	☐	For Bayesian analysis, information on the choice of priors and Markov chain Monte Carlo settings
☒	☐	For hierarchical and complex designs, identification of the appropriate level for tests and full reporting of outcomes
☒	☐	Estimates of effect sizes (e.g. Cohen's d, Pearson's r), indicating how they were calculated

Our web collection on statistics for biologists contains articles on many of the points above.

Software and code

Policy information about availability of computer code

Data collection	No software was used to collect data.
Data analysis	A detailed description of all open source software is provided in the Methods under the section 'Phylogenetic analysis and cluster generation'. This includes Trimmomatic v0.38, Kraken (v2.0.7), Snippy v4.3.5, Prokka v1.13.37, and NGmaster v0.5.5.

For manuscripts utilizing custom algorithms or software that are central to the research but not yet described in published literature, software must be made available to editors/reviewers. We strongly encourage code deposition in a community repository (e.g. GitHub). See the Nature Research guidelines for submitting code & software for further information.

Data

Policy information about availability of data

All manuscripts must include a data availability statement. This statement should provide the following information, where applicable:

- Accession codes, unique identifiers, or web links for publicly available datasets
- A list of figures that have associated raw data
- A description of any restrictions on data availability

All SRA data have been uploaded to BioProject PRJEB17738. Accession numbers are available in the Supplementary Data.

Field-specific reporting

Please select the one below that is the best fit for your research. If you are not sure, read the appropriate sections before making your selection.

Life sciences study design

All studies must disclose on these points even when the disclosure is negative.

Sample size	All available isolates in 2017 were included in the study, detailed in the Methods under the section 'Setting, patients and data sources.'
Data exclusions	There were no specific exclusions.
Replication	As is standard in our laboratory, phylogenetic analyses and results were verified by at least two individuals.
Randomization	Not relevant, as all samples were included.
Blinding	In order to ensure no bias was introduced into the interpretation of results, initial bioinformatic analysis was performed blinded to any metadata.

Reporting for specific materials, systems and methods

We require information from authors about some types of materials, experimental systems and methods used in many studies. Here, indicate whether each material, system or method listed is relevant to your study. If you are not sure if a list item applies to your research, read the appropriate section before selecting a response.

Materials & experimental systems

n/a	Involved in the study	
☒	☐	Antibodies
☒	☐	Eukaryotic cell lines
☒	☐	Palaeontology
☒	☐	Animals and other organisms
☐	☒	Human research participants
☒	☐	Clinical data

Methods

n/a	Involved in the study	
☒	☐	ChIP-seq
☒	☐	Flow cytometry
☒	☐	MRI-based neuroimaging

Human research participants

Policy information about studies involving human research participants

Population characteristics	Detailed information regarding the population included in the study is provided in the Methods, under the section 'Setting, patients and data sources', and in the Results section, under the heading 'Epidemiological characteristics of patients', and in Table 1. In total, 2,055 patients were included in the study, of which 86% were males, and 13% were females.
Recruitment	All patients who had a clinical sample that cultured Neisseria gonorrhoeae in the state of Victoria in 2017 were included.
Ethics oversight	Data in this study were collected under the Victorian Public Health and Wellbeing Act 2008. In addition, ethical approval was obtained from the Alfred Hospital Ethics Committee (Project 625/17). This information is also provided in the manuscript, and detailed information can be made available to reviewers on request.

Note that full information on the approval of the study protocol must also be provided in the manuscript.
