# Supplementary material for: Bridging of Neisseria gonorrhoeae lineages across sexual networks in the HIV pre-exposure prophylaxis era
Source: Nat Commun. 2019 Sep 5;10:3988. doi: 10.1038/s41467-019-12053-4 (PMC6728426; doi:10.1038/s41467-019-12053-4)
Supplement: Supplementary file 4 — Description of Additional Supplementary Files [file 41467_2019_12053_MOESM4_ESM.pdf]

Description of Additional Supplementary Files

File Name: Supplementary Data 1
Description: Metadata, accessions, and results for isolates used in this study

File Name: Supplementary Data 2
Description: Additional MLST 9363 strains used from the Gonococcal Isolate Surveillance Project
