# Supplementary material for: Bridging of Neisseria gonorrhoeae lineages across sexual networks in the HIV pre-exposure prophylaxis era
Source: Nat Commun. 2019 Sep 5;10:3988. doi: 10.1038/s41467-019-12053-4 (PMC6728426; doi:10.1038/s41467-019-12053-4)
Supplement: Supplementary file 2 — Peer Review File [file 41467_2019_12053_MOESM2_ESM.pdf]

Reviewers' Comments:

Reviewer #1:
Remarks to the Author:
This is a very comprehensive study analyzing NG isolates by WGS to determine and/or confirm that "bridging" between sexual networks occurs.

Here are some minor changes/edits to consider

1) The majority of isolates were collected from MSM. While the statistical analysis suggest a strong association between bridging of NG clones and populations subgroups, this reviewer feels that the low number of isolates, in comparison to MSM, from heterosexuals (men and women) should be listed as a limitation of the study.

2) Lines 127 - 128. The authors should provide more details on "our epidemiologically validate single-linkage 10 SNP threshold to define a possible transmission" Was this approach previously validate in another study.

3)Lines 142 - 147. While the presence of bisexual men and women in the major clusters may suggest bridging, is it also possible that some of the NG strains have been previously introduced to the heterosexual population without bridging from other sexual networks and instead from international travel. Was the data analyzed in this context? The present study only looks at data from 2017.

4)The NG displaying low-level azithromycin resistant represent an interesting group of isolates to further explore bridging between sexual networks. Did the authors determine if these isolates are unique to clusters were bridging occurred? Or are these isolates also present in clusters were heterosexual transmission is the only mode of transmission?

5)In terms of overseas travel, are any of those isolates from bisexual men which could be used to further illustrate bridging.

6) Lines 181 - 182. Was the mosaic mtr locus similar to that of N. meningitidis also presence in the isolates with high level resistance to Azithromycin.

7) Line 204 - 206. While this reviewer agrees that reducing the incidence of gonorrhea in MSM is a great strategy to reducing the incidence in other groups, a major emphasizes should be placed on bisexual men.

Minor comments.

1) Supplementary Figure 2. Please fix the age ranges on the X axis. They are not legible.

2) Figure 1 - Isolate site. Please add % of total

3) Figure 2 - Bottom box. This figure is hard to understand.

Reviewer #2:

Remarks to the Author:
The study analysed epidemiological data and genome sequences of more than 2000 Neisseria gonorrhoeae (Ng) isolates to understand Ng transmission between different subgroups-Men who have sex with Men, heterosexuals, biosexual MSM, HIV-positive MSM, HIV-negative PrEP users, and sex workers. The study identified bisexual men may play a role in bridging between different subgroups. The authors also reported an azithromycin resistance Ng clone circulating in MSM. Overall, the paper was well structured and well written. The reviewer only had a few comments listed below.

Comments

Lines 35-36. "bisexual MSM were identified as a possible 'bridging' population". Were there any epi links identified between bisexual men and females/MSM?

Lines 41-42. Not clear what hotspots mean here and how they can improve Ng control

Lines 104-106. "Compared to the 1,951 patients with only one infection, these 88 individuals were more likely to be MSM, HIV-positive, and PrEP users". Does this imply PrEP may not be that effective?

Lines 123-124. How was the SNP threshold (10 SNPs) determined? Has been validated using epidemiologically-linked pairs previously?

Lines 134-137. Determination of Associations requires statistical analysis.

Figure 1. Please indicate in legend that the numbers (at both end of the lines) in the bottom box are cluster number. Figure 1 shows isolates with less than 10 SNPs difference, but isolates in cluster 24 have >10 SNPs as shown in Figure 3.

Lines 142-146. You would expect these bridging cases occurred at early time within each cluster. When did these bridging cases occur?

All Figures and tables: Check the labels for all figures and tables; I saw several unusual symbols in figures and tables that may have resulted from conversion.

Reviewer #3:
Remarks to the Author:
This study uses whole genome sequencing of NG isolates in the Victoria state in Australia to provide descriptive information on demographics, sexual risk behaviors, HIV status, PrEP use, and AMR of NG transmission clusters. This information is potentially useful in understanding ongoing gonorrhea transmission patterns in this area in three specific ways: the bridging role that bisexual men play between homosexual/gay men and heterosexual women, the role of pharyngeal exposures in female sex workers, and the amplifying role that HIV PrEP may play in driving transmission. Unfortunately, this study featured only descriptive statistics, and so did not provide evidence to adequately answer of these three associational questions. Presumably (although not stated) this might have been because the sample size was quite low for the first two and PrEP use was dispersed across the transmission clusters for the last. So, the reader is left with vague conclusions that bisexuals bridge between MSM and heterosexuals (by definition true) and that PrEP use is common among MSM who have NG. In the end, this study provides interesting exploratory data for this specific time and geographic setting, but it is unclear what broader scientific conclusions may be drawn.

Specific Comments

1. The limitations of the descriptive nature of the study emerge most clearly with the analyses related to PrEP use. The dataset could potentially be quite useful to establish several associations of public health importance and scientific interest. These include whether transmission clusters are larger in PrEP users compared to non-PrEP users, or whether there would be evidence of more AMR in PrEP users. Instead the results of PrEP use prevalence are presented cluster-by-cluster ("Clusters 179 and 66 were particularly associated with… PrEP use amongst MSM…"), without clear associational measures. Relatedly, it would be of interest whether transmission chains could be established linking HIV-negative non-PrEP users with HIV-infected persons.

2. The use of the term "sexual networks", starting with the title and continuing throughout the paper, is confusing because they are never defined yet play a foundational role in the interpretation of the data. For example, in the abstract it is stated that widespread transmission of NG was identified "within and between sexual networks". Sexual networks here is primarily taken to mean inferred transmission events within clusters, but then it is unclear what "between" means in this context. Does this mean between persons of different individual-level demographic/risk attributes? Consistent definition of terms for population subgroups, transmission events, and clusters is needed.

3. The results are framed as finding evidence for direct transmission between groups, but that is limited by the low sampling fraction of diagnosed cases (28%) who had isolates available for analysis. It is also not mentioned that many cases that are undiagnosed, and that misclassification may be differential by attributes. Related to that, two-third of isolates were from MSM. It would be important to see the distribution of characteristics (Table 1) for all diagnosed cases versus isolates available to understand sampling biases. Relatedly, most of the data came from sexual health clinics. Is there potential for underreporting from primary care clinics oriented to women, and is there concern about low rates of NG diagnosis in women?

4. It is stated that the methods here could be used for gonorrhea interventions, but it is not clear what those interventions would be above the existing standard of care. Furthermore, it is unclear if the WGS method could or would realistically be employed in real-time to identify new clusters, as this analysis depended upon sequence data that was 1.5-2.5 years old. The specific public health utility of these methods should be clarified.

5. There are inconsistencies between risk behavior and sexual orientation in the paper. For example, the dataset contains information on sexual orientation in categories like "exclusively homosexual" but then the results are reported in categories defined behaviorally (e.g., men who have sex with men). Some of the presented results related to bridging therefore turn out to be true by definition (bisexuals must be the bridge between MSM and heterosexuals). For clarity, it would be better to use purely behavioral definitions if possible as these boundaries may cross (e.g., men who identify as heterosexual may be MSM).

6. It was unclear whether the variables that could be time-varying (HIV status, PrEP use, sexual behavior) preceded NG infection, complicating interpretations of directionality with transmission events.

Reviewer #1 (Remarks to the Author):

1. This is a very comprehensive study analyzing NG isolates by WGS to determine and/or confirm that "bridging" between sexual networks occurs.

We thank the Reviewer for this positive comment.

Here are some minor changes/edits to consider

2. The majority of isolates were collected from MSM. While the statistical analysis suggest a strong association between bridging of NG clones and population subgroups, this reviewer feels that the low number of isolates, in comparison to MSM, from heterosexuals (men and women) should be listed as a limitation of the study.

We agree with the reviewer and have added comments into manuscript to address this (Lines 253-270):

'A limitation of this work was the relatively low proportion of isolates included from females (13%) and heterosexual males (12%) compared to MSM (75%). It is likely that the lower proportion of N. gonorrhoeae isolates from females reflects the overall epidemiology of gonorrhoea in our setting - in 2017, females represented only 18.6% of all gonorrhoea notifications (i.e. culture plus NAAT) in Victoria.[32] This caveat is not unique to our study – for example, in a previous study assessing gonorrhoea transmission in London and Brighton in the UK, females comprised only 6% of the total dataset.[16] Similarly, in a recent WGS-based European study, only 15% of isolates were from females.[13] Further, approximately 10% of isolates in our study were from males with unknown sexual risk factors; it is possible that a proportion of these isolates were from heterosexual males. Future work should attempt to

increase the number of cultures obtained from females and heterosexual males to provide additional validation of our observations.'

3. Lines 127 - 128. The authors should provide more details on "our epidemiologically validate single-linkage 10 SNP threshold to define a possible transmission" Was this approach previously validated in another study.

Thank you, we agree this important point warrants further discussion, and have provided additional wording in the manuscript to address this (described in Lines 84-87 and 90-93). This approach was previously validated in a previous study (Kwong et al., Sex Transm Infect, 2018), and we have expanded on this in the paper (Lines 69-73 and 381-383).

4. Lines 142 - 147. While the presence of bisexual men and women in the major clusters may suggest bridging, is it also possible that some of the NG strains have been previously introduced to the heterosexual population without bridging from other sexual networks and instead from international travel. Was the data analyzed in this context? The present study only looks at data from 2017.

Thanks – we agree with this point, and have addressed this comment by performing additional analysis relating to international travel (expanded Supplementary Table 4). In addition, we have assessed the timing of introduction of isolates into clusters that are associated with international travel (new Supplementary Figure 8 and Lines 131-139 in Results). These additional analyses have shown that (i) overseas-acquired gonorrhoea is significantly more likely than locally-acquired gonorrhoea in heterosexual males, and (ii) of the 31 clusters containing travel-associated cases, the first isolate in 13 (42%) of these clusters came from a travel-associated case. We have expanded on these points considerably in the Discussion, including the potential public health implications of this finding (Lines 221-234).

4.The NG displaying low-level azithromycin resistant represent an interesting group of isolates to further explore bridging between sexual networks. Did the authors determine if these isolates are unique to clusters were bridging occurred? Or are these isolates also present in clusters were heterosexual transmission is the only mode of transmission?

Isolates with low-level azithromycin resistance were only present in clusters with MSM (either with or without heterosexual cases) – i.e. were not present in clusters with only heterosexual transmission. We agree this is an interesting group of isolates to explore further, and we have provided additional phylogenetic analysis on this group, by contextualising our data with recently available data from the United States (Thomas et al., J Infect Dis, 2019). This analysis demonstrates the global dissemination of a distinct low-level azithromycin-resistant lineage, belonging to MLST 9363, associated with mutations in the *mtr* locus, and circulating predominantly amongst MSM. We believe this is an important new and novel finding of the study, and we thank Reviewer #1 for this suggestion. We have provided additional information in Supplementary Figure 10, and in Lines 348-358 of the Methods, Lines 150-162 of the Results, and Lines 236-248 and Lines 279-282 of the Discussion.

5. In terms of overseas travel, are any of those isolates from bisexual men which could be used to further illustrate bridging.

Thanks for this suggestion. Only three isolates from bisexual males were identified as travel-associated, and all were non cluster-associated cases – i.e. not linked to any other cases.

6. Lines 181 - 182. Was the mosaic mtr locus similar to that of N. meningitidis also presence in the isolates with high level resistance to Azithromycin.

Neither of the two isolates with high-level azithromycin resistance had a mosaic *N.meningitidis*-like *mtr* locus. This information is available in the Supplementary Dataset, and we have now specifically mentioned this in the footnote to Supplementary Table 5.

7. Line 204 - 206. While this reviewer agrees that reducing the incidence of gonorrhoea in MSM is a great strategy to reducing the incidence in other groups, a major emphasizes should be placed on bisexual men.

We agree with this point, and have now made this explicit in the Discussion (Line 185). Further, we have also expanded considerably on the role of returning travellers as possible drivers for gonorrhoea transmission (Lines 221-234).

Minor comments.

8. Supplementary Figure 2. Please fix the age ranges on the X axis. They are not legible.

Thanks – this was a file conversion error, which we have amended.

9. Figure 1 - Isolate site. Please add % of total

Thanks – we have done this.

10. Figure 2 - Bottom box. This figure is hard to understand.

We have now expanded on the Figure legend to provide additional information relating to the Figure content, and also reviewed all figures to ensure they are easily readable.

Reviewer #2 (Remarks to the Author):

1. The study analysed epidemiological data and genome sequences of more than 2000 Neisseria gonorrhoeae (Ng) isolates to understand Ng transmission between different subgroups-Men who have sex with Men, heterosexuals, bisexual MSM, HIV-positive MSM, HIV-negative PrEP users, and sex workers. The study identified bisexual men may play a role in bridging between different subgroups. The authors also reported an azithromycin resistance Ng clone circulating in MSM. Overall, the paper was well structured and well written. The reviewer only had a few comments listed below.

We thank the Reviewer for this positive comment.

2. Lines 35-36. "bisexual MSM were identified as a possible 'bridging' population". Were there any epi links identified between bisexual men and females/MSM?

Contact tracing is not routinely performed on all cases of gonorrhoea in Victoria – as such we cannot say whether links between groups are direct or indirect. However, in the thirteen patient pairs who identified each other as contacts, there were no bisexual males.

3. Lines 41-42. Not clear what hotspots mean here and how they can improve Ng control

Thank you – we agree this wording around the key public health findings was not optimal, and have removed the word '*hotspots*.' We have also added wording in the Discussion around the potential public health implications of our findings (Lines 276 to 284)

4. Lines 104-106. "Compared to the 1,951 patients with only one infection, these 88 individuals were more likely to be MSM, HIV-positive, and PrEP users". Does this imply PrEP may not be that effective?

No – our observation suggests that individuals with any of these characteristics are more likely to have two or more infections. In a footnote to Supplementary Table 2, we have stated that we have excluded HIV-positive patients when assessing the number of infections amongst PrEP users. Our observation that reinfection is more common in PrEP users provides genomics-based evidence to support recent epidemiological studies assessing STI incidence in PrEP users (e.g. Traeger et al., JAMA 2019). We have now expanded on this in the Discussion (Lines 192 to 198). To further address Reviewer #2's comment, we have modified the wording in the manuscript to read: *'Compared to the 1,951 patients with only one infection, these 88 individuals with two or more infections were significantly more likely to be MSM, HIV-positive, or PrEP users (*Supplementary Table 2*).*

5. Lines 123-124. How was the SNP threshold (10 SNPs) determined? Has been validated using epidemiologically-linked pairs previously?

We agree this point warrants further discussion (and was also highlighted by Reviewer #1, point 3). We have expanded on this further in the Methods and Results (described in Lines 84-87 and 90-93), and provided information on the validation of our approach (Lines 69-73 and 381-383).

6. Lines 134-137. Determination of Associations requires statistical analysis.

We agree with this point and have added this information to Supplementary Table 3 and provided additional information in the Methods (Lines 385 -387).

7. Figure 1. Please indicate in legend that the numbers (at both end of the lines) in the bottom box are cluster number. Figure 1 shows isolates with less than 10 SNPs difference, but isolates in cluster 24 have >10 SNPs as shown in Figure 3.

Thanks – we have expanded the wording in the legend for Figure 1 (as per Reviewer #1) to provide additional information and have also expanded on the legend in Figure 3.

8. Lines 142-146. You would expect these bridging cases occurred at early time within each cluster. When did these bridging cases occur?

Thanks for this point. We believe that the opportunities for bridging may occur at any point during a cluster, depending on the activities and structure of a network of individuals. In the paper, we do not specifically state that bridging from bisexual males sustain heterosexual transmission clusters, only that they provide a potential bridging opportunity (Lines 110-112). To address Reviewer #2's comments, we have added some wording stating that we were unable

to determine whether isolates from bisexual males occurred at the start of clusters (Lines 174-177).

9. All Figures and tables: Check the labels for all figures and tables; I saw several unusual symbols in figures and tables that may have resulted from conversion.

Thanks – we have corrected this, and have checked all figures and tables for conversion errors.

Reviewer #3 (Remarks to the Author):

1. This study uses whole genome sequencing of NG isolates in the Victoria state in Australia to provide descriptive information on demographics, sexual risk behaviours, HIV status, PrEP use, and AMR of NG transmission clusters. This information is potentially useful in understanding ongoing gonorrhea transmission patterns in this area in three specific ways: the bridging role that bisexual men play between homosexual/gay men and heterosexual women, the role of pharyngeal exposures in female sex workers, and the amplifying role that HIV PrEP may play in driving transmission. Unfortunately, this study featured only descriptive statistics, and so did not provide evidence to adequately answer of these three associational questions. Presumably (although not stated) this might have been because the sample size was quite low for the first two and PrEP use was dispersed across the transmission clusters for the last. So, the reader is left with vague conclusions that bisexuals bridge between MSM and heterosexuals (by definition true) and that PrEP use is common among MSM who have NG. In the end, this study provides interesting exploratory data for this specific time and geographic setting, but it is unclear what broader scientific conclusions may be drawn.

We thank Reviewer #3 for their comments, and have addressed these below. We have added in additional analyses and discussion relating to PrEP use, overseas travel, and public health implications of our work. Further, (after additional biostatistical consultation), we have added in measures of statistical association with major clusters. We have also added a section on possible sampling bias (noting that this limitation is not unique to our study but applies to all genomics-based studies of *N. gonorrhoeae*). These inclusions are detailed below, and we believe have substantially improved the revised manuscript.

Specific Comments

2. The limitations of the descriptive nature of the study emerge most clearly with the analyses related to PrEP use. The dataset could potentially be quite useful to establish several associations of public health importance and scientific interest. These include whether transmission clusters are larger in PrEP users compared to non-PrEP users, or whether there would be evidence of more AMR in PrEP users. Instead the results of PrEP use prevalence are presented cluster-by-cluster ("Clusters 179 and 66 were particularly associated with… PrEP use amongst MSM…"), without clear associational measures. Relatedly, it would be of interest whether transmission chains could be established linking HIV-negative non-PrEP users with HIV-infected persons.

We agree with these comments. To address them, we have provided new analyses of (i) cluster size in PrEP users compared to non-PrEP users (Supplementary Figure 7A), and (ii) a timeline of all transmission clusters that include PrEP users (Supplementary Figure 7B). These analyses

show that the median size of clusters in PrEP users is significantly larger than those associated with non-PrEP users, and that there are transmission networks associated with HIV-infected and HIV-non-infected individuals (Supplementary Figure 7B). Further, after biostatistical consultation, we have added statistical associations to Supplementary Table 3. We have taken the opportunity to considerably expand upon these findings in the Discussion, with specific mention of the possible public health relevance of these findings (Lines 192-198 and Lines 276-284). We believe these additions provide important and novel genomics-based insights into PrEP use and gonorrhoea transmission, and we thank Reviewer #3 for these suggestions.

3. The use of the term "sexual networks", starting with the title and continuing throughout the paper, is confusing because they are never defined yet play a foundational role in the interpretation of the data. For example, in the abstract it is stated that widespread transmission of NG was identified "within and between sexual networks". Sexual networks here is primarily taken to mean inferred transmission events within clusters, but then it is unclear what "between" means in this context. Does this mean between persons of different individual-level demographic/risk attributes? Consistent definition of terms for population subgroups, transmission events, and clusters is needed.

Thank you for this suggestion – we agree with this and have standardised terminology from 'sexual networks' to 'population risk groups' and 'transmission clusters' throughout, and have provided additional information in the Results used to define a transmission event, and how transmission clusters were generated (Lines 84-92)

4. The results are framed as finding evidence for direct transmission between groups, but that is limited by the low sampling fraction of diagnosed cases (28%) who had isolates available for analysis. It is also not mentioned that many cases that are undiagnosed, and that misclassification may be differential by attributes. Related to that, two-third of isolates were from MSM. It would be important to see the distribution of characteristics (Table 1) for all diagnosed cases versus isolates available to understand sampling biases. Relatedly, most of the data came from sexual health clinics. Is there potential for underreporting from primary care clinics oriented to women, and is there concern about low rates of NG diagnosis in women?

As per response to Reviewer #1 (point 2), this sampling bias is not unique to our study, and is a broader reflection of the complexity of gonorrhoea epidemiology, the lower proportion of disease in women and heterosexual males, and a general decline in culture-based testing for *N. gonorrhoeae*. However, we have provided additional wording to reflect this in the revised manuscript (Lines 45-47 and Lines 253-264). We note that, while the distribution of subgroups in our study may not exactly match the overall distribution of subgroups notified with gonorrhoea, this does not detract from the fact we have identified bridging of distinct lineages across population subgroups - which we believe is the more important finding. However, to specifically address Reviewer #3's comment, we have taken the opportunity to highlight the need for increased *N. gonorrhoeae* cultures from females and heterosexual males in future genomics-based studies (Lines 263-264).

5. It is stated that the methods here could be used for gonorrhea interventions, but it is not clear what those interventions would be above the existing standard of care. Furthermore, it is unclear if the WGS method could or would realistically be employed in real-time to identify new clusters, as this analysis depended upon sequence data that was 1.5-2.5 years old. The specific public health utility of these methods should be clarified.

Thank you for raising this point regarding clarity of the public health implications of the technology. To address this, we have added a new section in the Discussion which details the potential public health implications of our study (Lines 276-288). These include enhanced advice to travellers, regular screening of sex workers and PrEP users, and identification of broader risk networks associated with genetic clusters. Importantly, these interventions are not likely to be the 'existing standard of care' in all settings.

Given the routine implementation of WGS into public health microbiology in many settings, including for high volume diseases such as salmonellosis, it is realistic that a WGS-based genomic surveillance approach could be used in real-time to identify new clusters. This is highlighted by a recent study on the real-time use of WGS to respond to an outbreak of high-level azithromycin-resistant *N. gonorrhoeae* (Fifer et al., Lancet ID, 2018). While academic publications may follow many months and sometimes years after WGS is generated, public health actions can be implemented as new data is generated and analysed.

6. There are inconsistencies between risk behavior and sexual orientation in the paper. For example, the dataset contains information on sexual orientation in categories like "exclusively homosexual" but then the results are reported in categories defined behaviorally (e.g., men who have sex with men). Some of the presented results related to bridging therefore turn out to be true by definition (bisexuals must be the bridge between MSM and heterosexuals). For clarity, it would be better to use purely behavioral definitions if possible as these boundaries may cross (e.g., men who identify as heterosexual may be MSM).

Thank you for this suggestion. To address this comment, we have amended our terminology to behavioural definitions, rather than sexual orientation. We have used 'men who have sex with men' throughout, and where relevant, have subclassified this into 'men who have sex with men only' (MSMO) to represent individuals who identified as exclusively homosexual, and 'men who have sex with men and women' (MSMW) to represent individuals who identified as bisexual (Lines 309-310, and changed throughout the text). We have also added additional details in Table 1 and Supplementary Table 3.

7. It was unclear whether the variables that could be time-varying (HIV status, PrEP use, sexual behavior) preceded NG infection, complicating interpretations of directionality with transmission events.

Thank you for this comment, and we agree that this is a difficult area (not unique to our study). However, we specifically do not mention directionality of transmission in our paper, but rather assess the temporal spread of transmission clusters.

Reviewers' Comments:

Reviewer #1:
Remarks to the Author:
Thanks to the authors for making the requested changes and additional analysis.

Please consider make this following minor changes.

Line 63 - 65. "Compared to the 1,951 patients with only one infection, these 88 individuals with two or more infections were significantly more likely to be MSM, HIV-positive, or PrEP users (Supplementary Table 2". Please add p-value at the end of the sentence

Line 65 - 68. "Of the 16 patients whose isolates were cultured ≥14 days apart, but differed by less than 100 SNPs, nine patients had repeat isolates that belonged to the same transmission cluster as their initial isolate (median duration between isolates days, range 18-201 days". It would be interesting to confirm that this is not associated with treatment failure. Since susceptibility data is available, this reviewer suggests looking at the susceptibility data of these isolates and to report, if accurate, that these isolates represent a new infection.

Line 141 - "Further, isolates from patients acquiring their infections overseas were more likely to be resistant to penicillin, tetracycline and ciprofloxacin, and less resistant to azithromycin compared to patients with locally-acquired infections (Supplementary Table 4)" Please add p-value.

Reviewer #3:
Remarks to the Author:
Revision addresses all major concerns from initial review.

REVIEWERS' COMMENTS

Reviewer #1

Thanks to the authors for making the requested changes and additional analysis. Please consider make this following minor changes.

1. Line 63 - 65. "Compared to the 1,951 patients with only one infection, these 88 individuals with two or more infections were significantly more likely to be MSM, HIV-positive, or PrEP users (Supplementary Table 2". Please add p-value at the end of the sentence.

We have added in P values for the respective characteristics.

2. Line 65 - 68. "Of the 16 patients whose isolates were cultured ≥14 days apart, but differed by less than 100 SNPs, nine patients had repeat isolates that belonged to the same transmission cluster as their initial isolate (median duration between isolates days, range 18-201 days". It would be interesting to confirm that this is not associated with treatment failure. Since susceptibility data is available, this reviewer suggests looking at the susceptibility data of these isolates and to report, if accurate, that these isolates represent a new infection.

Thanks – all nine patients had isolates with the same susceptibility profile – as such, we feel that reporting of this data is not essential, and would prefer to base our assumptions on our genomic analysis.

3. Line 141 - "Further, isolates from patients acquiring their infections overseas were more likely to be resistant to penicillin, tetracycline and ciprofloxacin, and less resistant to azithromycin compared to patients with locally-acquired infections (Supplementary Table 4)" Please add p-value.

Thanks – we have added this, but are happy to defer to copy editors whether this is essential or whether it is sufficient to reference the Table.

Reviewer #3

Revision addresses all major concerns from initial review.

Thanks again for reviewing our paper.
